# *METTL23* mutation alters histone H3R17 methylation in normal-tension glaucoma

**DOI:** 10.1172/JCI153589

**Published:** 2022-11-01

**Authors:** Yang Pan, Akiko Suga, Itaru Kimura, Chojiro Kimura, Yuriko Minegishi, Mao Nakayama, Kazutoshi Yoshitake, Daisuke Iejima, Naoko Minematsu, Megumi Yamamoto, Fumihiko Mabuchi, Mitsuko Takamoto, Yukihiro Shiga, Makoto Araie, Kenji Kashiwagi, Makoto Aihara, Toru Nakazawa, Takeshi Iwata

**Affiliations:** 1 National Institute of Sensory Organs, National Hospital Organization Tokyo Medical Center, Tokyo, Japan.; 2 Department of Ophthalmology, Tokai University Hachioji Hospital, Tokyo, Japan.; 3 Kimura Eye Clinic, Iwaki, Japan.; 4 Cancer Precision Medicine Center, Japanese Foundation for Cancer Research, Tokyo, Japan.; 5 JAC Ltd., Tokyo, Japan.; 6 Department of Ophthalmology, University of Yamanashi, Yamanashi, Japan.; 7 Department of Ophthalmology, University of Tokyo, Tokyo, Japan.; 8 Department of Ophthalmology, Tohoku University Graduate School of Medicine, Sendai, Japan.; 9 Kanto Central Hospital of the Mutual Aid Association of Public School Teachers, Tokyo, Japan.

**Keywords:** Ophthalmology, Genetic diseases, Mouse models, iPS cells

## Abstract

Normal-tension glaucoma (NTG) is a heterogeneous disease characterized by retinal ganglion cell (RGC) death leading to cupping of the optic nerve head and visual field loss at normal intraocular pressure (IOP). The pathogenesis of NTG remains unclear. Here, we describe a single nucleotide mutation in exon 2 of the methyltransferase-like 23 *(METTL23)* gene identified in 3 generations of a Japanese family with NTG. This mutation caused *METTL23* mRNA aberrant splicing, which abolished normal protein production and altered subcellular localization. *Mettl23*–knock-in (*Mettl23^+/G^* and *Mettl23^G/G^*) and -knockout (*Mettl23^+/–^* and *Mettl23^–/–^*) mice developed a glaucoma phenotype without elevated IOP. METTL23 is a histone arginine methyltransferase expressed in murine and macaque RGCs. However, the novel mutation reduced METTL23 expression in RGCs of *Mettl23^G/G^* mice, which recapitulated both clinical and biological phenotypes. Moreover, our findings demonstrated that METTL23 catalyzed the dimethylation of H3R17 in the retina and was required for the transcription of *pS2*, an estrogen receptor α target gene that was critical for RGC homeostasis through the negative regulation of NF-κB–mediated TNF-α and IL-1β feedback. These findings suggest an etiologic role of METTL23 in NTG with tissue-specific pathology.

## Graphical abstract

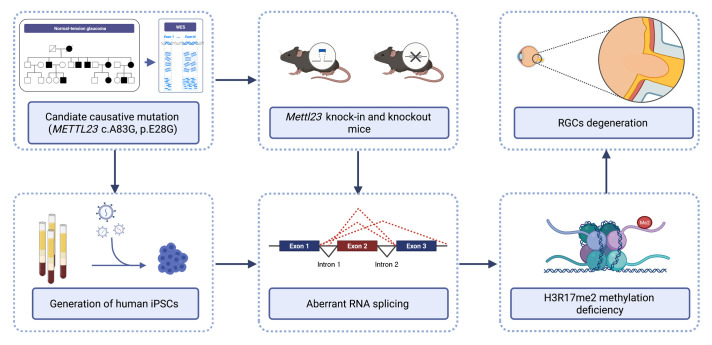

## Introduction

Glaucoma is the most frequent cause of progressive and irreversible blindness and is characterized by optic nerve head cupping and visual field loss (1). Open-angle glaucoma is the most prevalent glaucoma subtype (2). The Baltimore Eye Survey reported that over half of the patients with open-angle glaucoma do not have elevated intraocular pressure (IOP) (3), thus meeting the case definition of normal-tension glaucoma (NTG). Epidemiologic studies have reported a higher NTG prevalence among Asians (52%–92%), especially among Japanese people with NTG (92%) compared with White people (30%–39%) (4–7), and that Asian Americans had a 159% increased risk of NTG compared with non-Hispanic White individuals (8), possibly reflecting an increased demographic association and genetic susceptibility. Moreover, 21% of patients with NTG have a family history of glaucoma (9), further suggesting a genetic predisposition. The optineurin (OPTN) E50K mutation and TANK-binding protein 1 *(TBK1)* copy number variations are responsible for familial NTG with autosomal dominant inheritance (2, 10–15). OPTN negatively regulates the transcription factor NF-κB, which is expressed ubiquitously and is crucial for cell proliferation, antiapoptosis, and the immune response (16, 17). TBK1 also attenuates NF-κB activation by degrading one of the IκB kinases, NF-κB–inducing kinase (18, 19). Moreover, the OPTN E50K mutation increases TBK1-OPTN binding (20, 21), potentially enhancing NF-κB activity and promoting apoptosis (22). However, mutations in these 2 genes account for only 2%–3% of NTG cases (11), suggesting that additional genes may contribute to NTG by encoding proteins involved in NF-κB signaling.

Methylation is one of the most important posttranslational modifications of histones and plays a vital role in regulating gene expression and transcription and, in turn, is implicated in various disease processes (23–25). There are 2 categories of enzymes involved in histone methylation based on target residues: protein lysine methyltransferases and protein arginine methyltransferases (23). Arginine methylation is a prevalent modification that displays tissue-specific functions (26). Methyltransferase-like 23 (METTL23) has distant homology with protein arginine methyltransferases and catalyzes the asymmetric dimethylation of histone H3R17 in murine oocytes (27). However, its enzymatic activities in other tissues and in disease have not been evaluated.

This study describes, for the first time to our knowledge, an association of histone methylation with glaucoma. A number of researchers have predicted epigenetics involvement in glaucoma, which we believe the present study proves. Here, we performed whole-exome sequencing (WES) of 11 individuals including 6 patients from a 3-generation family with NTG. As a result, we identified the *METTL23* c.A83G variant as a pathogenic mutation. We assessed its effects on expression, localization, and methylation by splicing using 3 transfected cell lines, patient-derived induced pluripotent stem cells (iPSCs), and *METTL23* c.A83G–knock-in (KI) mice. We also determined in vivo whether *METTL23* defects cause retinal ganglion cell (RGC) degeneration and optic nerve head fiber loss, the typical human NTG phenotype, and explored the role of METTL23 in methylation in vitro and in vivo.

## Results

### Clinical presentation.

**Figure 1:**
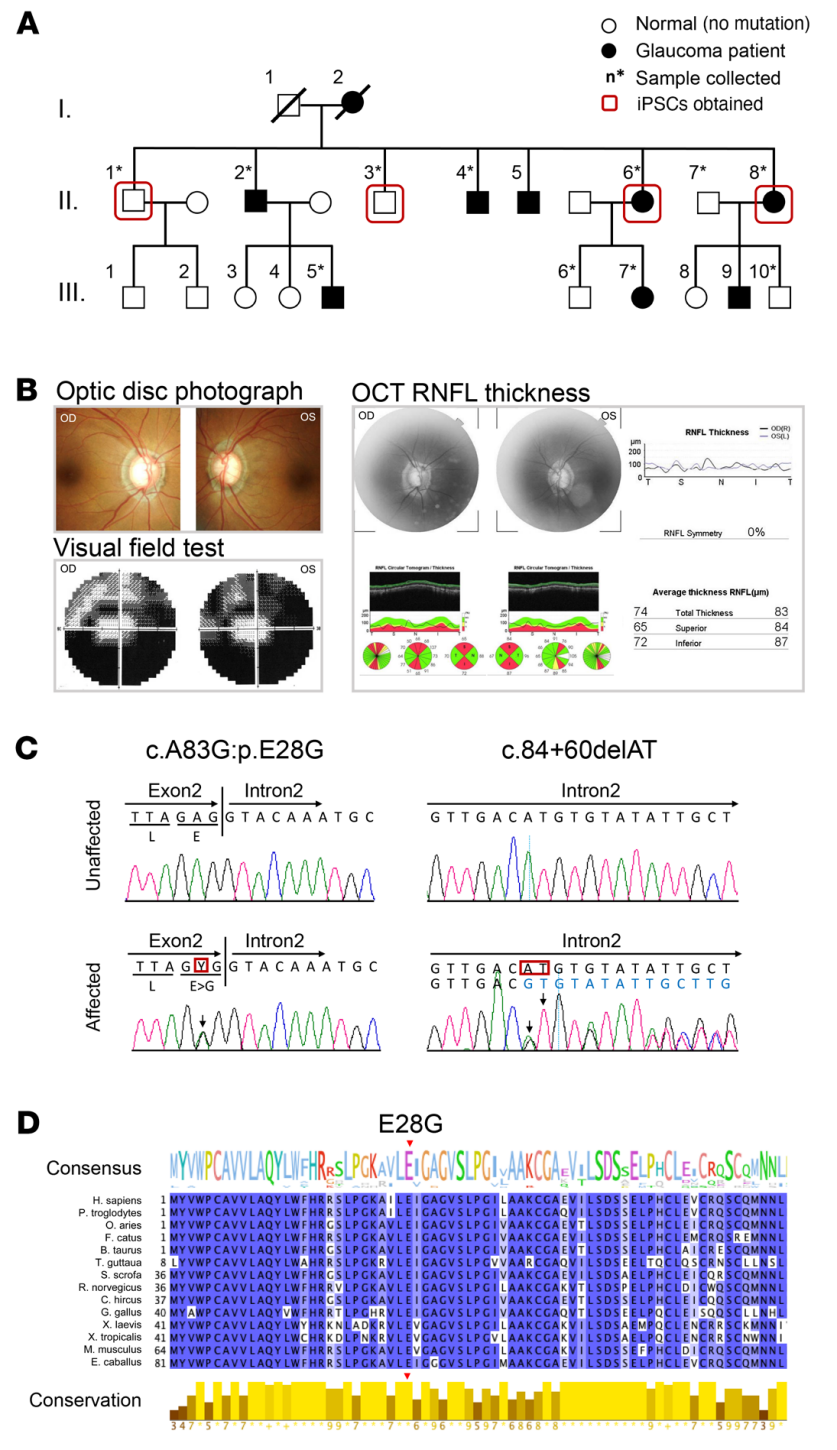
Identification of the *METTL23* c.A83G mutation. (**A**) Pedigree of the Japanese family with members with NTG. Affected individuals are indicated by solid black symbols. Collected samples are marked by asterisks. (**B**) Clinical manifestations of a patient with NTG (**A**, patient II-8). OD, oculus dexter; OS, oculus sinister. (**C**) Sanger sequencing chromatogram of the c.A83G (p.E28G) mutation indicated by the red box at the end of exon 2. (**D**) Subfamily-specific conservation. The variant’s position is shown above. Histograms show the degree of conservation at each residue.

We identified a large, dominant inheritance NTG pedigree of Japanese origin in 9 affected individuals (Figure 1A). The clinical findings are summarized in Supplemental Table 1 (supplemental material available online with this article; https://doi.org/10.1172/JCI153589DS1). None of the affected members had a history of developmental delay or retinal disease. Physical examinations revealed loss of the neuroretinal rim and widening of the optic cup in the presence of normal IOP (Figure 1B and Supplemental Figure 1). Moreover, visual field testing results from NTG patient II-8 showed progressive defects over 8 years of follow-up (Figure 1B and Supplemental Figure 2).

### Identification of candidate causative mutations.

To identify novel NTG-causing genes, we performed WES data analysis on 6 affected and 5 unaffected individuals from the family with NTG of unknown molecular etiology (Supplemental Table 2). Compared with the reference human genome (hs37d5), 19,624 missense mutations were detected in the affected individuals. By following nucleotide variations that cause common amino acid substitutions and that appear at low frequency (<1%) in public databases (Exome Aggregation Consortium [ExAC], Genome Aggregation Database [gnomAD], Human Genetic Variation Database [HGVD], 4.7KJPN) and in our in-house database, we identified 5 variants as candidates. Next, we filtered the remaining variants using the inheritance pattern with sibling DNA and identified *METTL23* and centrosomal protein 290 (*CEP290*) as the putative disease-causing genes. Functional prediction by Pholyphen2, Sorting Intolerant From Tolerant (SIFT), and Protein Variation Effect Analyzer (PROVEAN) identified *METTL23* p.E28G (c.A83G) (RefSeq ID: NM_001080510) as the candidate pathogenic variant, whereas the *CEP290* variant was excluded because of its low pathogenicity score (Table 1, Figure 1C, and Supplemental Table 3). The in silico predictive tool Human Splicing Finder (HSF) (3.1, http://www.umd.be/HSF3/HSF.html) indicated that the splice donor mutation in *METTL23* (c.A83G) causes abnormal splicing and results in functionally null proteins (Table 2). Additionally, according to an analysis of evolutionary conservation (Figure 1D and Supplemental Figure 3; Jalview), computational structure (Supplemental Figure 4, A and B; PHYRE2, http://www.sbg.bio.ic.ac.uk/~phyre2/html/page.cgi?id=index), and multiple sequence alignment of METTL23 with homologs of known 3D structure including METTL21A, METTL21B, METTL21C, and METTL21D (Supplemental Figure 4, C and D, and Supplemental Table 4; Jalview and JPred4, http://www.compbio.dundee.ac.uk/jpred4/index.html), the *METTL23* c.A83G:p.E28G mutation, which is involved in motif 1, is expected to affect protein function and is not found in any other patients with glaucoma enrolled in the Japan Whole Exome Project. Read depth data for each of the *METTL23* exons were extracted from the patients’ exomes and compared with the average read depth. There were no additional exon-spanning duplications or deletions in any of the patients. Furthermore, no *TBK1* copy number variations were detected (Supplemental Figure 5A).

### Conserved METTL23 c.A83G mutation exhibits gain of splicing in vitro.

**Figure 2:**
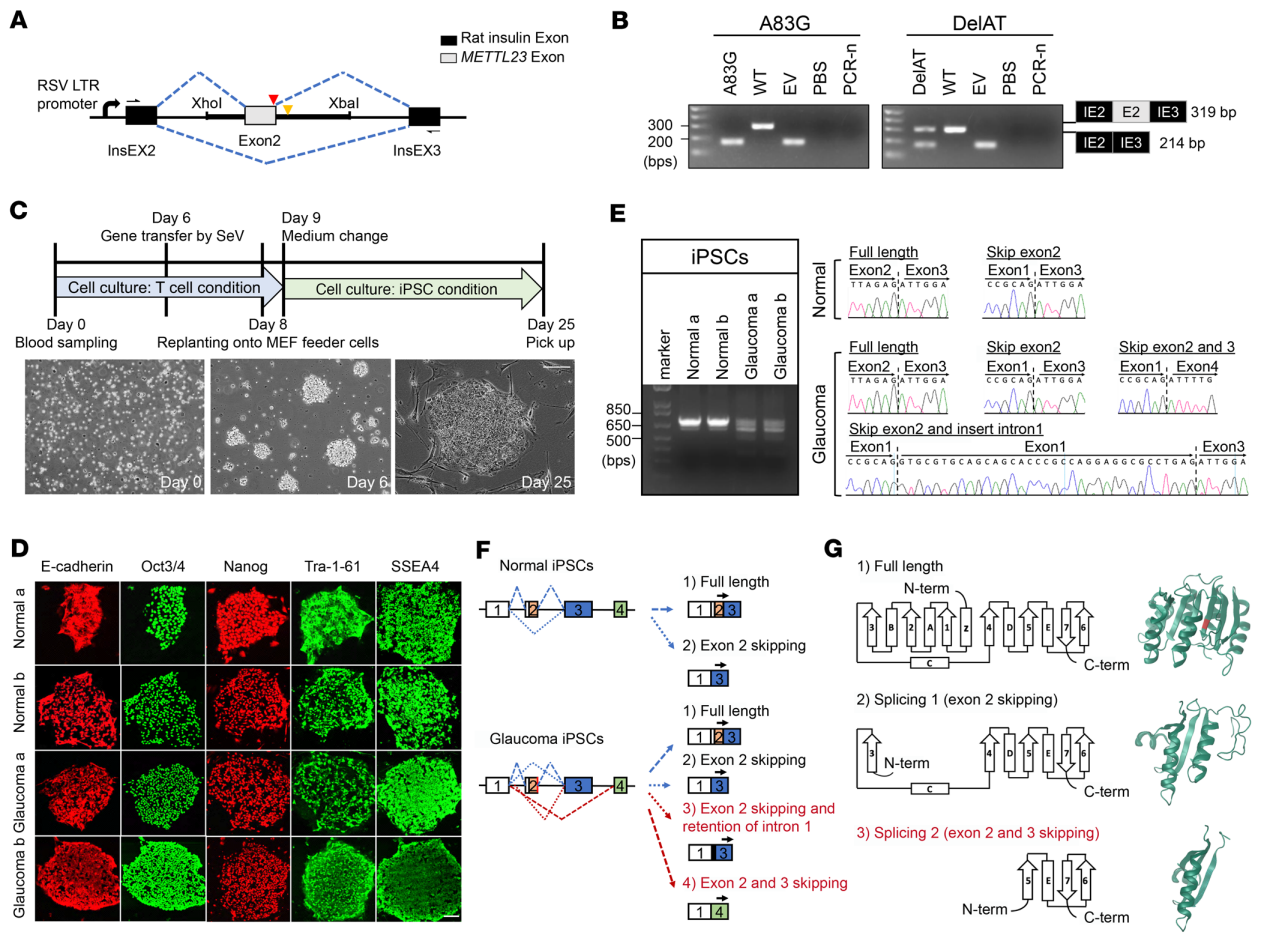
The *METTL23* c.A83G mutation leads to splicing in transfected HEK293T cells and iPSCs. (**A**) The splicing construct minigene was generated by incorporating the genomic region of the *METTL23* gene into the pSpliceExpress vector via XhoI and XbaI restriction sites. Vector exons are depicted as black boxes, and the *METTL23* exon 2 is shown as a gray box. The locations of the mutations are marked by arrowheads (red: c.A83G; yellow: c.84+60delAT). (**B**) Gel electrophoresis of RT-PCR products from transfected HEK293T cells. The primers are indicated by arrows in **A**. EV, empty vector; PBS, cells transfected with PBS only; PCR-n, PCR negative control. WT and mutant transcript contents were determined by Sanger sequencing and are depicted to the right of the gel image. (**C**) Graphic display of iPSC preparation. Collected samples are marked with a red box in Figure 1A. Scale bar: 200 μm. (**D**) Characterization of iPSCs. A83G-iPSC colonies (glaucoma a and b) were derived from lymphocytes from patients with NTG. There was no difference between iPSC controls (normal a/b) and A83G-iPSCs (glaucoma a/b) during induction and cultivation. Scale bar: 200 μm. (**E**) Gel electrophoresis of RT-PCR products from iPSCs. TA-cloning analysis following Sanger sequencing identified 4 splicing variants in A83G-iPSCs (glaucoma a/b). (**F**) Schematic representation of the splicing variants. (**G**) Predicted protein structures of the splicing variants (red: p.E28G). The 2 predicted proteins lack motif 1 and motif post 1, suggesting that the mutant alleles are functionally null. C-term, C-terminal; N-term, N-terminal.

To determine the effect of *METTL23* c.A83G on mRNA splicing, we tested its functional impact by a splicing assay using HEK293T cells. In this system, the c.A83G splice donor site mutation resulted in complete skipping of exon 2, effectively removing 106 bp including the start codon from the mRNA transcript, by which amino acids 1–67 of METTL23 would not be translated (Figure 2, A and B). Moreover, we confirmed *METTL23* c.A83G splicing in iPSCs from 2 patients with NTG (Figure 1A, patients II-6 and II-8). iPSCs were generated using peripheral blood lymphocytes from the patients carrying the c.A83G mutation and maintained pluripotency as evidenced by the expression of typical pluripotent stem cell markers (Figure 2, C and D, and Supplemental Figure 6). We observed complex aberrant RNA splicing in *METTL23* c.A83G iPSCs by TA cloning following Sanger sequencing (Figure 2E). In normal iPSCs derived from family controls, sequencing results showed only 2 mRNA variants: full-length and exon 2 deletion. However, in c.A83G iPSCs, we identified 2 additional novel mRNA splicing variants. The first skipped exons 2 and 3, while the other skipped exon 2 and inserted the initial 35 bp of intron 1 (Figure 2, E–G). Consequently, METTL23 would likely be inactive by lacking residues 1–28 or 1–107 aa, including motif 1, post 1, motif 2, and DXXY. As these motifs are critical for seven-β-strand methyltransferase function (28), we propose that the c.A83G mutation represents a loss-of-function mutation by splicing.

### Conserved METTL23 c.A83G mutation exhibits gain of splicing in vivo.

**Figure 3:**
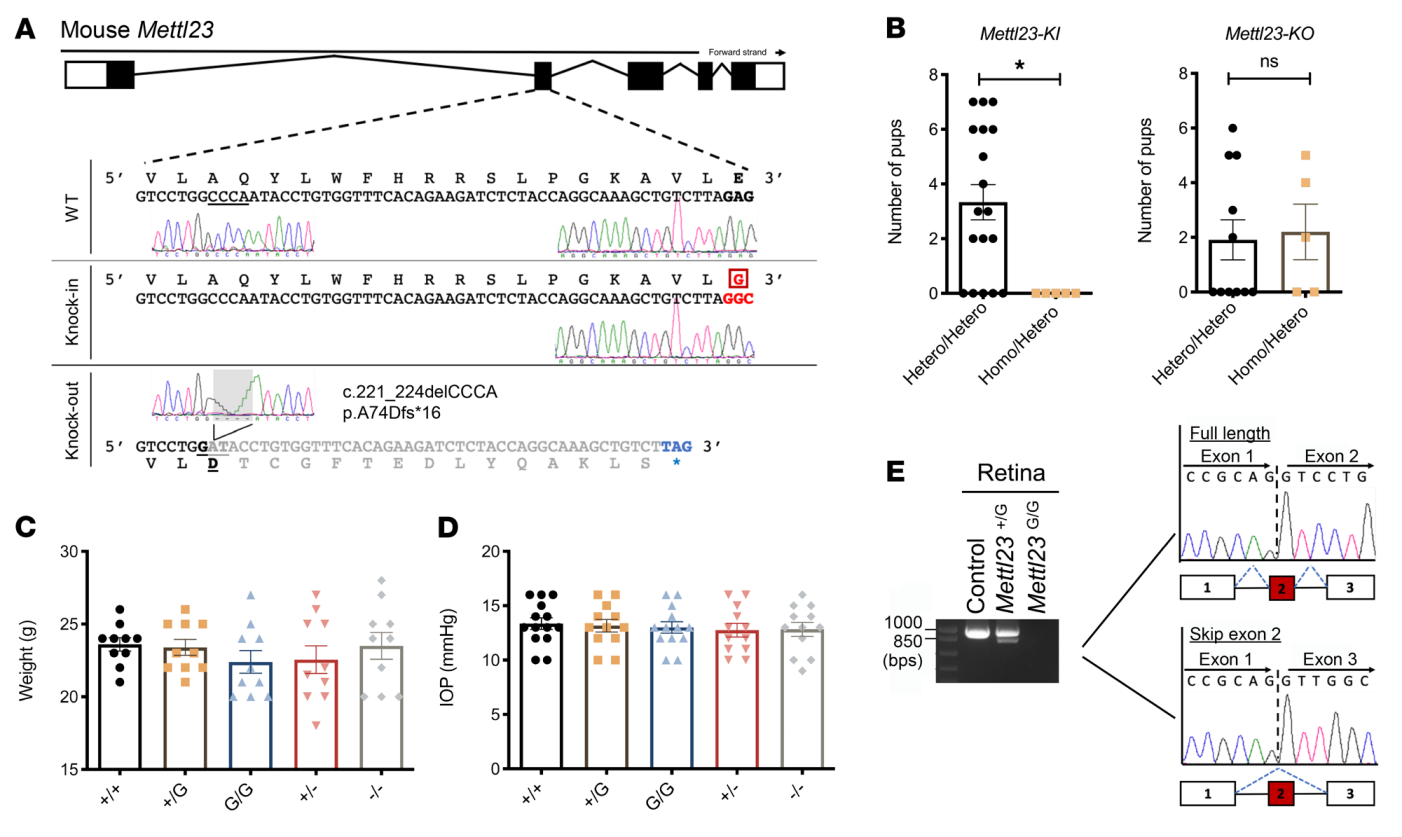
The *Mettl23* mutation causes exon skipping in vivo. (**A**) Sequence of the targeted region in *Mettl23*. *Mettl23*-KI and -KO mice were produced by genome editing using the CRISPR/Cas9 system. The mutant sequence is boxed in red. The STOP codon is indicated by blue letters. (**B**) Number of pups born after natural mating of *Mettl23*-KI or -KO mice. Hetero/Hetero, mating of heterozygous pairs of mice; Homo/Hetero, mating of homozygous and heterozygous mice. Dots represent the number of pups per litter on the day of birth. (**C**) Body weights of WT (*n* = 10), *Mettl23*-KI *Mettl23^+/G^* (*n* = 10), *Mettl23^G/G^* (*n* = 10), *Mettl23^+/–^* (*n* = 10), and *Mettl23^–/–^* mice (*n* = 10). At 2 months of age, all *Mettl23* genetic mice had body weights within the normal range. (**D**) IOPs for WT (*n* = 14), *Mettl23^+/G^* (*n* = 12), *Mettl23^G/G^* (*n* = 13), *Mettl23^+/–^* (*n* = 12), and *Mettl23^–/–^* (*n* = 12) mice. At 2 months of age, all mice were examined under identical conditions and exhibited IOPs within the normal range. (**E**) Gel electrophoresis of RT-PCR products from retinas of *Mettl23^+/G^* and *Mettl23^G/G^* mice. The contents of the application products were determined by TA cloning following Sanger sequencing. All data are presented as the mean ± SEM. *P < 0.05, by Student’s *t* test.

Furthermore, we investigated the splicing impact of the glaucoma-causing mutation in KI mice that carried the identical mutation *Mettl23 c.A83G* produced by a CRISPR/Cas9 system (Figure 3A). Mice carrying the mutation were born alive, and the mutation was confirmed by DNA-Seq of tail specimens (Figure 3A). Except for litter sizes (Figure 3B), heterozygous KI (*Mettl23^+/G^*) and homozygous KI (*Mettl23^G/G^*) mice were indistinguishable from controls in gross appearance, weight (Figure 3C), and IOP (Figure 3D). cDNA fragments amplified from murine retinas by *Mettl23* primers were cloned into TA vectors. The splice donor site mutation led to complete skipping of exon 2 in *Mettl23^+/G^* mice and loss of *Mettl23* mRNA transcripts in *Mettl23^G/G^* mice (Figure 3E). Taken together, we reason that the glaucoma-causing *METTL23* mutation evokes loss of function because its spliced mRNAs lack at least 1 functional motif, or as a result of the abolishment of translation (Supplemental Figure 7).

### METTL23 c.A83G mutation leads to aberrant expression and abnormal intracellular localization.

**Figure 4:**
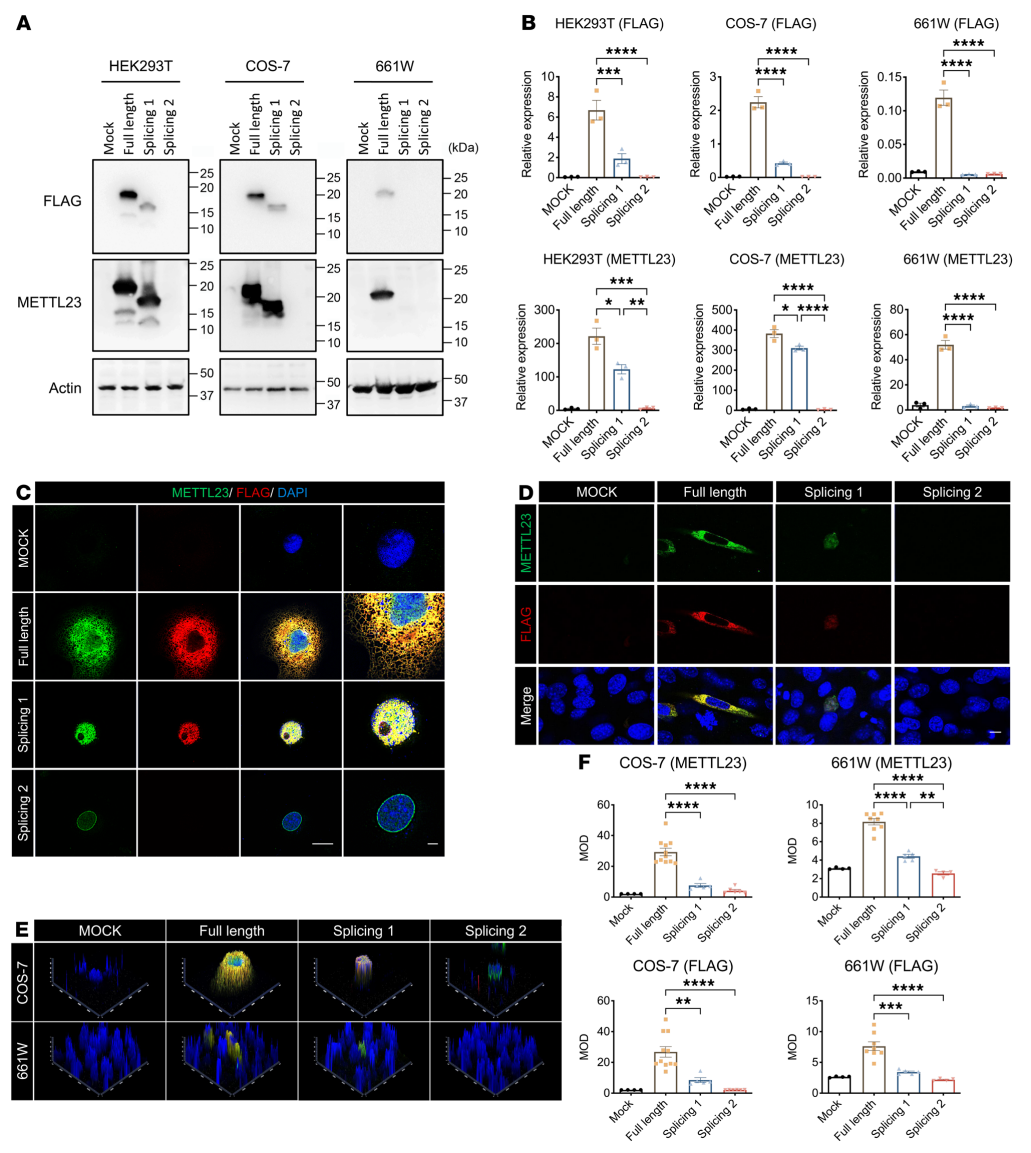
The *METTL23* c.A83G mutation leads to aberrant expression and trafficking in vitro. (**A**) METTL23 expression was reduced by the c.A83G mutation. Expression of full-length METTL23, splicing 1 (skip exon 2) and splicing 2 (skip exons 2 and 3) with the C-terminal FLAG-tag in HEK293T, COS-7, and 661W cells was determined by WB. (**B**) Quantification of METTL23 expression. Data are representative of 3 independent experiments. (**C** and **D**) Localization of METTL23 and different splicing products with the C-terminal FLAG-tag in transfected COS-7 (**C**) and 661W (**D**) cells by immunofluorescence. METTL23 was labeled with anti-METTL23 (green) and anti-FLAG (red) antibodies. Nuclei were stained with DAPI (blue). Scale bars: 10 μm. (**E**) 2.5D expression pattern of variant METTL23 in COS-7 and 661W cells. (**F**) Medium optical density (MOD) statistical results for METTL23 in transfected COS-7 and 661W cells. Results are representative of 4–11 independent experiments. All data are presented as the mean ± SEM. *P < 0.05, **P < 0.01, ***P < 0.001, and ****P < 0.0001, by 1-way ANOVA followed by Tukey’s multiple-comparison test.

To determine the effect of splicing variants on METTL23 protein production and intracellular trafficking, we generated a pEF-BOS-METTL23-FLAG expression vector and tested functionality by Western blotting (WB) and immunofluorescence. The splicing 1 (skip exon 2) and splicing 2 (skip exons 2 and 3) variants resulted in drastic reductions in METTL23 expression in transfected HEK293T, COS-7, and 661W cells (Figure 4, A and B). Because the subcellular localization of METTL23 is still unknown, we expressed METTL23 transiently with a C-terminal FLAG-tag in COS-7 and 661W cells. Immunofluorescence studies of COS-7 cells revealed that exogenous full-length METTL23 was present in typical endoplasmic reticulum and nuclear structures, while the splicing 1 variant was localized in the nucleoplasm, and the splicing 2 variant was localized in the nuclear membrane with reduced density (Figure 4, C, E, and F). Similarly, in transfected 661W cells, splicing induced the transfer of METTL23 from the cytoplasm to the nucleus (Figure 4, D–F).

### METTL23 localization in the retina.

**Figure 5:**
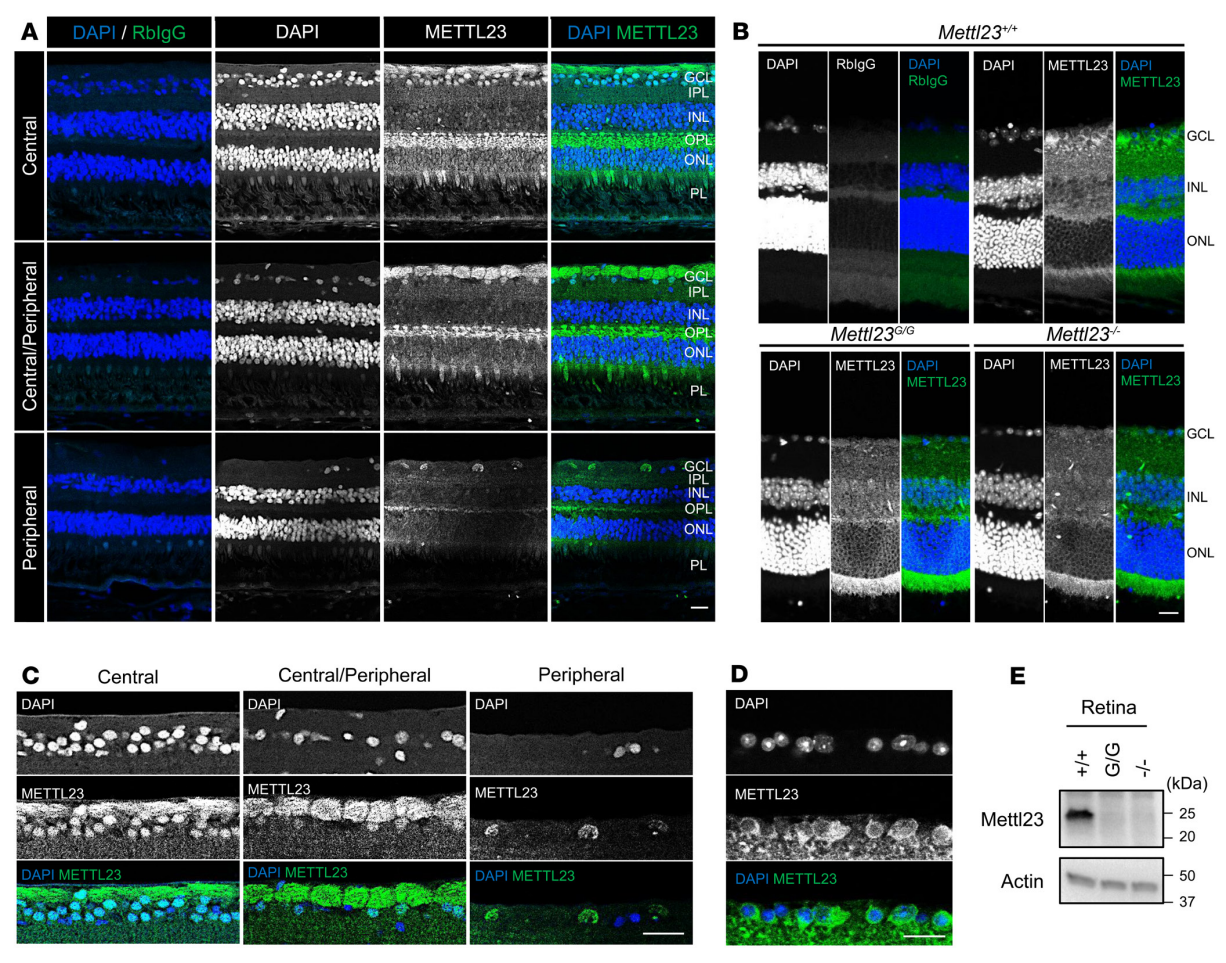
The *Mettl23* mutation reduces expression in vivo. (**A**) Localization of METTL23 in cynomolgus macaque retina. METTL23 was specifically expressed in RGC nuclei and optic nerve fibers, as demonstrated by immunofluorescence. Nuclei were stained with DAPI (blue) in the same section. The merged image reveals nuclear colocalization of METTL23. METTL23 expression was more intense in the central retina than in the periphery. (**B**) WT murine retina shows cytoplasmic and nuclear staining in RGCs with anti-METTL23 antibody (green) by immunofluorescence. In *Mettl23^G/G^* and *Mettl23^–/–^* mice, METTL23 lost specific RGC binding. (**C** and **D**) Higher-magnification images show a high degree of colocalization of METTL23 and RGCs in macaque (**C**, higher-magnification view of GCL in **A**) and murine (**D**) retinal tissue. (**E**) *Mettl23^G/G^* and *Mettl23^–/–^* mice lost retinal METTL23 expression as shown by WB. Scale bars: 20 μm. INL, inner nuclear layer; OPL, outer plexiform layer; ONL, outer nuclear layer; PL, photoreceptor layer.

We next investigated the endogenous retinal expression of METTL23. Retinal immunofluorescence revealed strong METTL23 expression in RGCs of both mice and cynomolgus macaques. In paraffin sections of macaque retinas, METTL23 was highly expressed specifically in RGC nuclei and optic nerve fibers (Figure 5, A and C), whereas in frozen sections of mouse retina, METTL23 was additionally expressed in RGC cytoplasm (Figure 5, B and D). In agreement with cell-based assays, immunofluorescence showed that METTL23 expression was markedly reduced in the RGCs of *Mettl23^G/G^* mice (Figure 5B). Furthermore, METTL23 protein was undetectable by WB in the retinas of *Mettl23^G/G^* mice (Figure 5E and Supplemental Figure 8). Collectively, our results demonstrated that the *METTL23* c.A83G mutation led to the loss of functional protein by the absence of protein production and altered subcellular localization.

### METTL23 c.A83G mutation and deficiency result in NTG.

**Figure 6:**
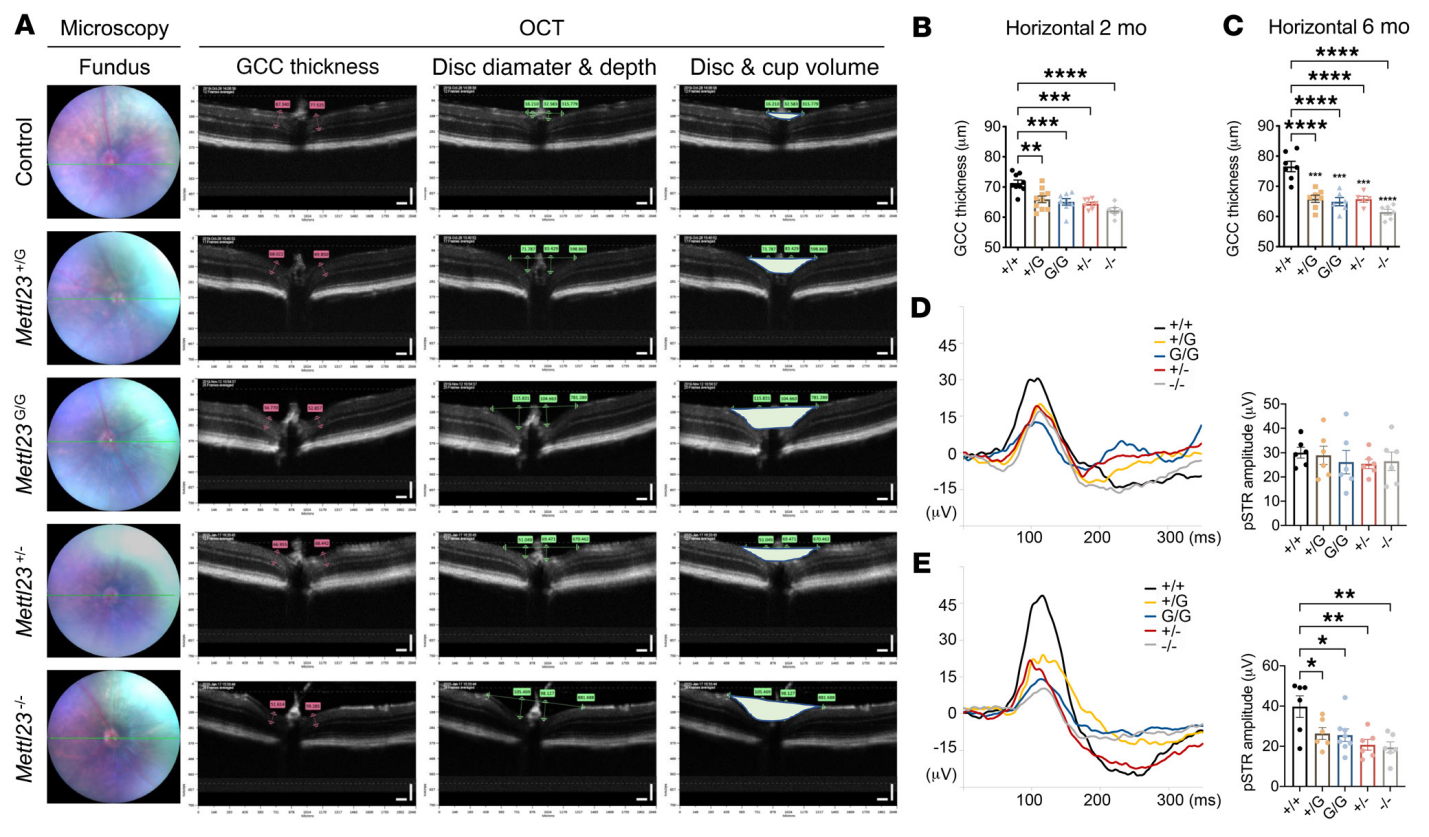
*Mettl23* mutation and deficiency cause morphologic and functional changes in RGCs by OCT and pSTR. (**A**) Representative OCT data were obtained from *Mettl23*-KI, -KO, and control mice by a B-horizontal scan centered on the optic nerve head. (**B** and **C**) Peripapillary GCC thicknesses in RGCs of *Mettl23^+/G^*, *Mettl23^G/G^*, *Mettl23^+/–^*, and *Mettl23^–/–^* mice were measured with Insight (Phoenix) and compared with thicknesses in RGCs of *Mettl23^+/+^* mice at 2 months (**B**) and 6 months (**C**) of age (*n* ≥6 per group). (**D** and **E**) pSTR of scotopic ERG in *Mettl23^+/+^, Mettl23^+/G^*, *Mettl23^G/G^*, *Mettl23^+/–^*, and *Mettl23^–/–^* mice. Histogram of pSTR peak amplitude changes in mice at 2 months (**D**) and 6 months (**E**) of age (*n* ≥6). Light intensity = –4.5 log scot cd·s/m^2^. All data are presented as the mean ± SEM. *P < 0.05, **P < 0.01, ***P < 0.001, and ****P < 0.0001, by 1-way ANOVA followed by Tukey’s multiple-comparison test. The GCC comprises the RNFL, GCL, and IPL.

To gain further insight into METTL23 function in the progressive degeneration of RGCs, we first analyzed the thickness of ganglion cell complexes (GCCs) (including the retinal nerve fiber layer [RNFL], the ganglion cell layer [GCL], and the inner plexiform layer [IPL]) and positive scotopic threshold response (pSTR) amplitudes in *Mettl2*3-KI and -KO mice compared with controls by ocular coherence tomography (OCT) and electroretinography (ERG), respectively. *Mettl23*-KO mice were generated with a deletion mutation, c.221_224del (A74fs), as determined by the absence of METTL23 protein in homozygous mutants (Figure 5E). *Mettl23^+/–^* and *Mettl23^–/–^* mice had normal weights (Figure 3C), IOPs (Figure 3D), and litter sizes (Figure 3B). In B-circular scans centered on the optic nerve head, we observed a reduction of GCC thickness in 2-month-old *Mettl23^–/–^* mice (Supplemental Figure 9, A and C) that had progressed in all *Mettl23*-KI and -KO mice by 6 months of age (Supplemental Figure 9, B and D). Similarly, B-horizontal images of OCT scans revealed significantly decreased GCC thickness in *Mettl23^+/G^*, *Mettl23^G/G^*, *Mettl23^+/–^*, and *Mettl23^–/–^* mice compared with controls at 2 months (Figure 6, A and B) and 6 months of age (Figure 6C). Moreover, the difference increased with age (Supplemental Figure 9, E and F), suggesting a progressive axonal loss, similar to the pathology observed in patients with glaucoma. We then assessed the functional activity of RGCs from *Mettl23^+/G^*, *Mettl23^G/G^*, *Mettl23^+/–^*, and *Mettl23^–/–^* mice noninvasively by the pSTR, which originates from the functional inner retina and reflects RGC activity (29). Slight reductions in pSTR amplitudes were observed in *Mettl23*-KI and -KO mice at 2 months of age (Figure 6D). *Mettl23^+/G^*, *Mettl23^G/G^*, *Mettl23^+/–^*, and *Mettl23^–/–^* mice developed significant RGC deficiencies at 6 months of age (Figure 6E).

**Figure 7:**
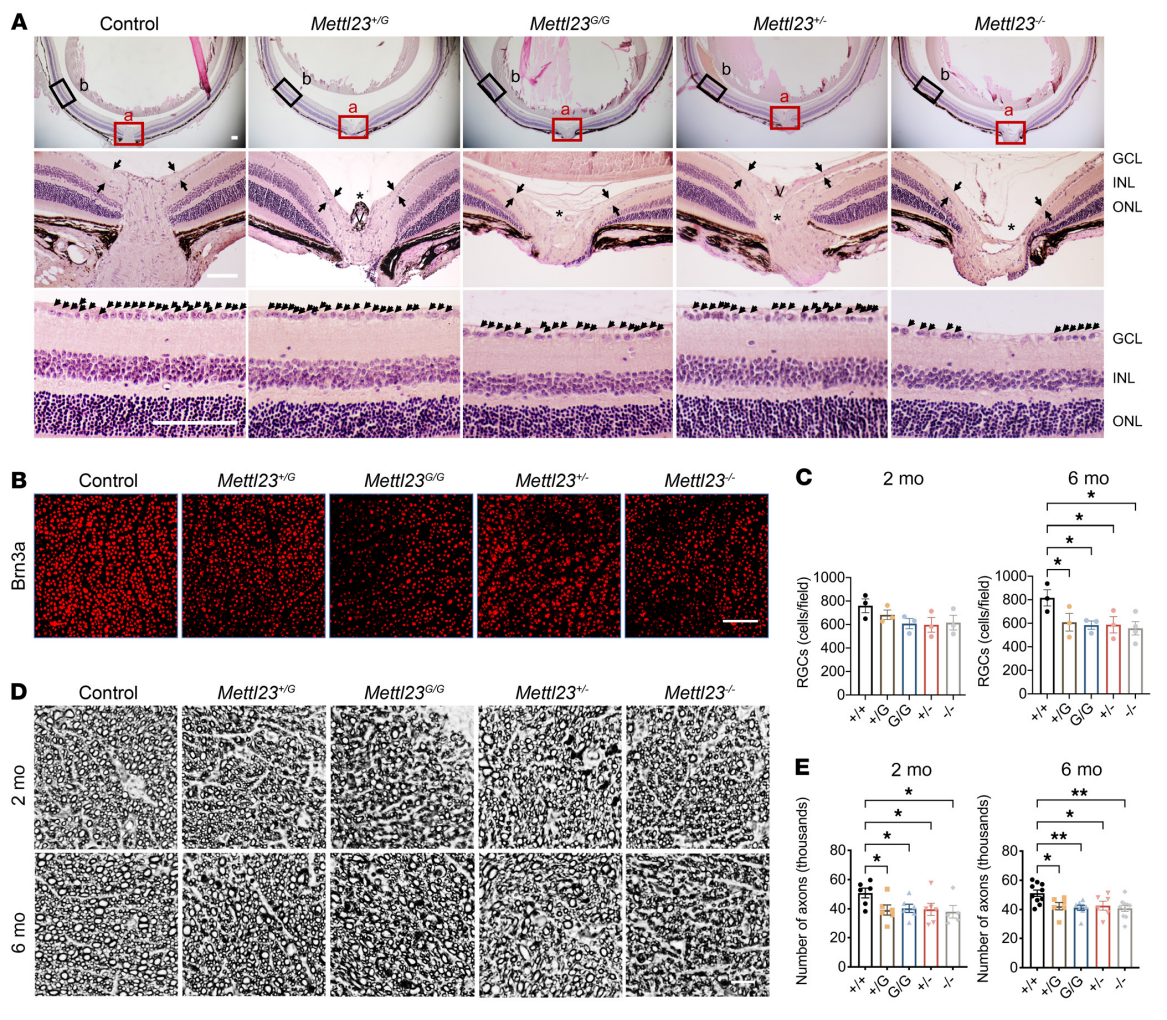
*Mettl23* mutation and deficiency cause RGC degeneration. (**A**) H&E staining of murine retinal sections from *Mettl23^+/G^*, *Mettl23^G/G^*, *Mettl23^+/–^*, *Mettl23^–/–^*, and control mice at 2 months of age. Scale bars: 100 μm. (**B**) Confocal images of whole-mount retinas with Brn3a-labeled RGCs from*Mettl23^+/G^*, *Mettl23^G/G^*, *Mettl23^+/–^*, *Mettl23^–/–^*, and control mice at 6 months of age. All images were taken from the mid-periphery of the retina. Scale bar: 100 μm. (**C**) RGC quantification was performed in a 700 × 700 μm area in 4 quadrants from the mid-periphery of the retina and averaged for mice at 2 and 6 months of age (*n* ≥3 retinas per group). (**D**) Representative cross-sectional images of optic nerve stained with paraphenylenediamine (PDD) from *Mettl23^+/G^*, *Mettl23^G/G^*, *Mettl23^+/–^*, *Mettl23^–/–^*, and control mice at 2 months and 6 months of age. Scale bar: 10 μm. (**E**) Quantification of axons stained with PDD in optic nerve cross-sections from *Mettl23^+/G^*, *Mettl23^G/G^*, *Mettl23^+/–^*, *Mettl23^–/–^*, and control mice at 2 and 6 months of age (*n* ≥6 retinas per group). All data are presented as the mean ± SEM. *P < 0.05 and **P < 0.01, by 1-way ANOVA followed by Tukey’s multiple-comparison test.

We further analyzed morphologic changes in histologic sections of *Mettl23^+/G^*, *Mettl23^G/G^*, *Mettl23^+/–^*, and *Mettl23^–/–^* mice. H&E-stained retinas showed severe optic nerve atrophy, including RGC loss, optic nerve fiber injury, and excitative remodeling of the optic nerve at 2 months (Figure 7A). To evaluate RGC loss in *Mettl23*-KI and -KO mice, we immunostained retinal whole mounts with the selective RGC marker Brn3a (30). RGCs were counted and averaged per 4 selected fields of identical size. Quantification revealed slight but insignificant decreases of RGCs in both *Mettl23*-KI and -KO mice compared with controls at 2 months of age (Figure 7C), which progressed to significant RGC loss at 6 months of age (Figure 7, B and C). To assess the effect of METTL23 on optic nerve fiber injury, we performed paraphenylenediamine staining, which labels the axonal myelin sheath (31), in optic nerve cross-sections (Figure 7D). We observed statistically significant differences in both *Mettl23*-KI and -KO mice compared with controls at 2 months and 6 months of age (Figure 7D and E). Consequently, these data support progressive RGC degeneration in *Mettl2*3-KI and -KO models, beginning with axonal injury that preceded neuronal loss. Notably, the affected individuals of the NTG family also developed glaucoma with normal IOP, suggesting a role for *Mettl23*-KI and -KO murine models in studying certain aspects of human glaucoma.

### METTL23 regulates H3R17me2a methylation in the retina.

**Figure 8:**
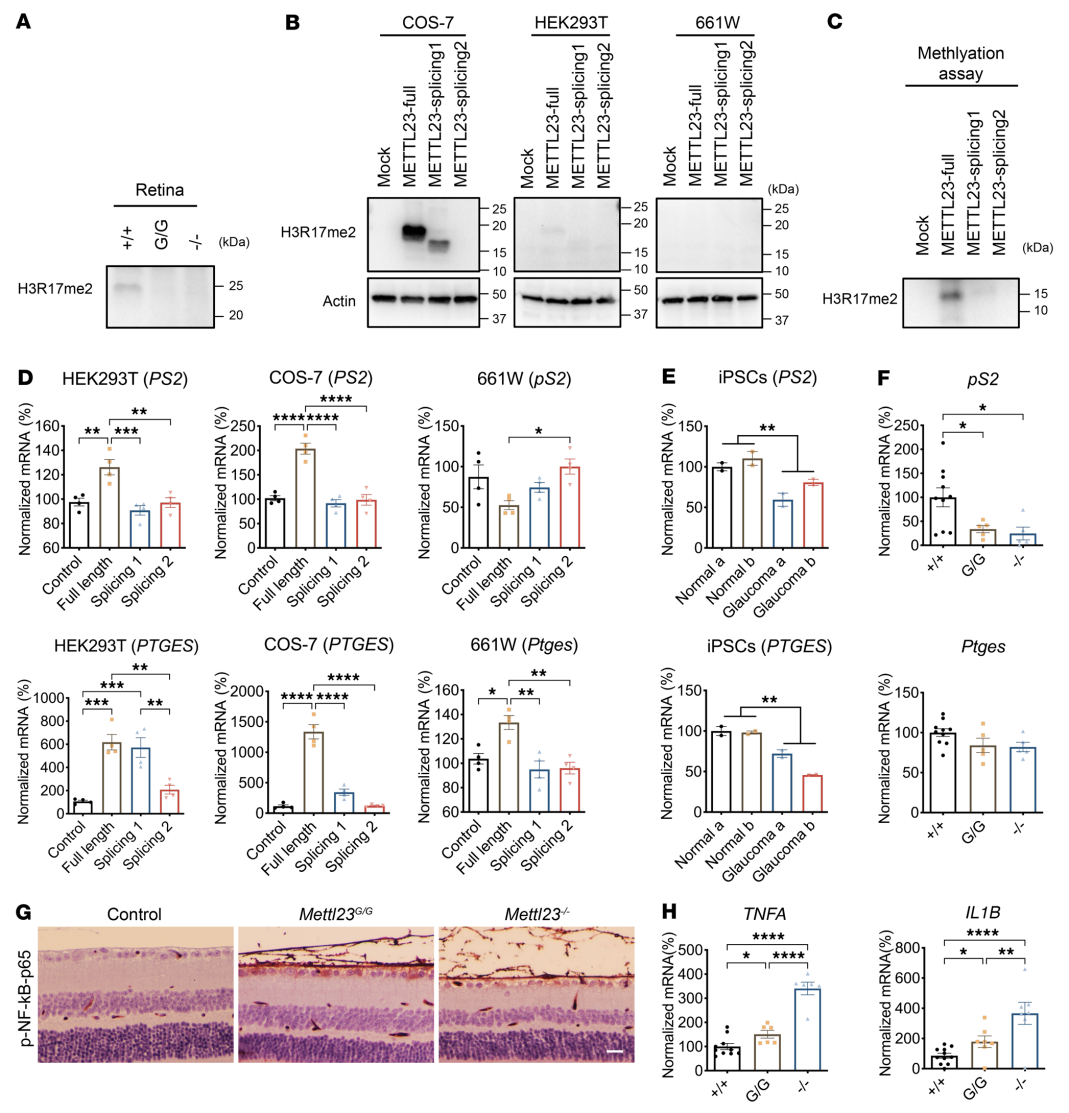
Methylation and regulation activity of METTL23 in vitro and vivo. (**A**) The *Mettl23* mutation reduced the methylation activity at arginine 17 of histone H3 (H3R17me2a) in the retina in vivo by WB. (**B**) The effect of METTL23 on arginine methylation in transfected cells. HEK293T, COS-7, and 661W cells were transfected with full-length METTL23 (METTL23-full), splicing 1, and splicing 2 in parallel. WB shows that dimethylation at arginine 17 of histone H3 (H3R17me2a) was induced in METTL23-full transfected cells using the specific antibody. (**C**) The effect of METTL23 mutation on arginine methylation through an in vitro methylation assay is shown by WB. METTL23-full-FLAG-His6, METTL23-splicing1-FLAG-His6 and METTL23-splicing2-FLAG-His6 were purified from transfected HEK293T cells, and WB was performed after an in vitro methylation assay. H3R17me2a was only induced by full-length METTL23. (**D**) A strengthening of the transcription of *PS2* and *PTGES*, 2 ERα target genes, was observed in METTL23-overexpressing cells. HEK293T, COS-7, and 661W cell lines were transfected with full-length METTL23, splicing 1, and splicing 2, respectively. The mRNA levels of *PS2*/*pS2* and *PTGES*/*Ptges* were analyzed by RT-qPCR, relative to *GAPDH* and *Gapdh* in quadruplicate. The attenuation of their transcription was observed in iPSCs derived from patients with NTG (**E**) and from retinas of *Mettl2*3*^G/G^* (*n* = 5) and *Mettl23*^–/–^ (*n* = 5) mice (**F**), compared with controls (*n* = 10). (**G**) Immunohistochemical staining for p–NF-κB–p65 (Ser539) in retinal sections from *Mettl2*3*^G/G^* and *Mettl23^–/–^* mice at 2 months of age. Scale bar: 20 μm. (**H**) mRNA levels of *TNFA* and *IL1B* were increased in *Mettl23^G/G^* (*n* = 6) and *Mettl23^–/–^* mice (*n* = 6) compared with levels in WT mice (*n* = 10). All data are presented as the mean ± SEM. *P < 0.05, **P < 0.01, ***P < 0.001, and ****P < 0.0001, by 1-way ANOVA followed by Tukey’s multiple-comparison test.

METTL23 catalyzes the asymmetric dimethylation of arginine 17 in histone H3 (H3R17me2a) in vitro and in murine oocytes (27), but its functional role in the retina is unknown. To demonstrate the retinal function of METTL23, we investigated its methylation activity in the murine retina by WB using an anti-H3R17me2a antibody. We found that METTL23 lost its H3R17me2a methylation activity in the retinas of *Mettl23^G/G^* and *Mettl23^–/–^* mice compared with age-matched controls (Figure 8A). The high degree of conservation between murine and human exons (Supplemental Figure 3) suggests that METTL23 is likely to catalyze H3R17me2a in the human retina.

We then demonstrated the methylation activity of *METTL23* c.A83G splicing products in vitro by WB. METTL23-splicing 1/2 expression vectors were transfected into COS-7 and HEK293T cells, respectively, and markedly reduced H3R17me2a methylation (Figure 8B). In transfected 661W cells, the H3R17me2a WB band was undetectable. This finding may have been due to low transfection efficiency; or, alternatively, the 661W cell line may not be representative of cells in which methylation is regulated by METTL23 (Figure 8B). To confirm the loss of methylation function by METTL23-splicing 1/2, we performed an in vitro methylation assay in the presence of histone H3.1 using METTL23-full–FLAG–His6 or METTL23-splicing 1/2–FLAG–His6 purified from transfected HEK293T cells. We found a substantial reduction in H3R17me2a levels in the presence of METTL23-splicing 1 or -splicing 2 compared with METTL23-full by WB using an anti-H3R17me2a antibody (Figure 8C). These studies clearly indicated that METTL23 catalyzes H3R17 dimethylation in the murine retina and that the novel mutation reduced its methylation activity.

### Loss of METTL23 increases TNF-α and IL-1β transcription in murine retina.

Coactivator-associated arginine methyltransferase 1 (CARM1) upregulates 5 estrogen receptor α (ERα) target genes by increasing the H3R17me2a mark in vitro (32, 33). We examined whether these genes were also regulated by METTL23 using reverse transcription quantitative real-time PCR (RT-qPCR) analysis. In transfected HEK293T and COS-7 cells, expression of the transcripts of both trefoil factor 1 (*PS2,* also called *TFF1)* and prostaglandin E synthase (*PTGES*) was significantly induced by overexpression of METTL23-full, but not by METTL23–splicing 1 or –splicing 2 (Figure 8D). METTL23 overexpression in transfected 661W cells enhanced the transcription of *Ptges* but not *pS2* (Figure 8D). Moreover, the transcription of *PS2*/*pS2* and *PTGES*/*Ptges* was significantly reduced in NTG patient–derived iPSCs and in *Mettl23^G/G^* and *Mettl23^–/–^* mice compared with controls (Figure 8, E and F). However, the transcription of *MYC*, *EGRS*, and *IGEBP4* was not regulated by METTL23 in vitro or in vivo (Supplemental Figure 10, A–L). Thus, METTL23 upregulated the transcription of *PS2* and *PTGES*, 2 of the ERα target genes, by increasing the H3R17me2a mark in vitro and in vivo.

NF-κB transcription factors are important in regulating the immune response (34, 35), while PS2 suppresses the NF-κB–activated expression of the proinflammatory cytokines TNF-α and IL-1β (36, 37). Increased TNF-α and IL-1β expression by activated microglial cells in hypoxic neonatal retina induced RGC apoptosis (38, 39). We first investigated whether METTL23 deficiency upregulates NF-κB activity in the retina of *Mettl23*-KI and -KO mice. Immunohistochemical staining for phosphorylated NF-κB–p65 (p–NF-κB–p65), which regulates the transcriptional activity of NF-κB (40), revealed intense immunostaining in the RGC and RNFLs of *Mettl23*-KI and -KO mice, but not in those of control mice (Figure 8G). We next examined the impact of loss of METTL23 on *TNFA* and *IL1B* transcription in murine retina. RT-qPCR results showed significantly increased expression of *TNFA* and *IL1B* mRNA in both *Mettl23^G/G^* and *Mettl23^–/–^* mice compared with controls (Figure 8H). Taken together, the results of immunostaining and RT-qPCR assays indicated that loss of METTL23 led to RGC degeneration by increasing NF-κB–mediated *TNFA* and *IL1B* transcription.

### METTL23 c.A83G mutation disrupts METTL23 expression, H3R17 dimethylation, and PS2 transcription in human iPSC-differentiated RGCs.

**Figure 9:**
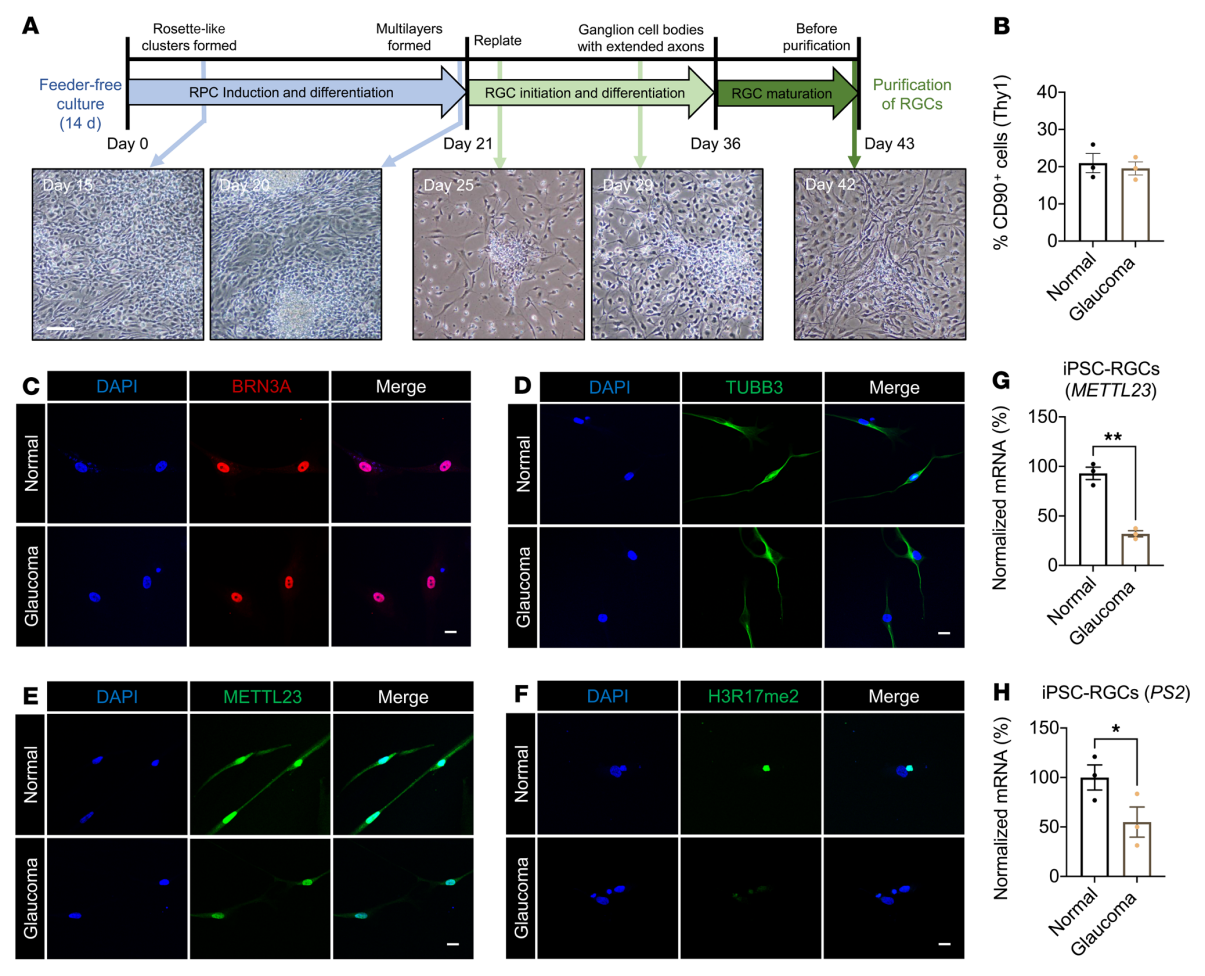
Characterization of iPSC-RGCs. (**A**) Graphic display of iPSC-RGC differentiation and purification. Scale bar: 100 μm. (**B**) Quantification of the percentage of CD90^+^ cells (*n* = 3, control: 20.96% ± 2.60%; glaucoma: 19.54% ± 1.75%). Immunofluorescence images of iPSC-RGCs induced by BRN3A (**C**) and TUBB3 (**D**). An immunofluorescence assay was performed to detect the expression of METTL23 (**E**) and H3R17 methylation (**F**) in iPSC-RGCs. Significant decreases were observed in METTL23 expression and H3R17 methylation in iPSC-RGCs derived from patients with glaucoma with the *METTL23* c.A83G mutation compared with controls. Scale bars: 20 μm (**C**–**F**). mRNA levels of *METTL23* (**G**) and *PS2* (**H**) were analyzed by RT-qPCR, relative to *GAPDH,* in triplicate. All data are presented as the mean ± SEM. *P < 0.05 and **P < 0.01, by Student’s *t* test.

To validate the effect of *METTL23* c.A83G on the physiological characterization of human RGCs, we induced the differentiation of iPSCs into RGCs (Figure 9A). Similar proportions of cells expressing CD-90, which is a surface glycoprotein uniquely expressed in RGCs (30), were generated from *METTL23* c.A83G (19.54% ± 1.75%) and control (20.96% ± 2.60%) iPSC lines, respectively (Figure 9B). To further investigate the identification of iPSC-differentiated RGCs (iPSC-RGCs), CD-90^+^ cells were then immunostained with BRN3A, TUBB3, and DAPI to show nuclei. The selected cells were labeled by both antibodies, indicating complete conversion (Figure 9, C and D). Notably, METTL23 expression, H3R17 dimethylation, and *PS2* transcription were reduced in *METTL23* c.A83G iPSC-RGCs compared with controls (Figure 9, E–H), suggesting that METTL23 was a positive modulator of H3R17 dimethylation and *PS2* transcription in RGCs. However, no significant differences were observed in NF-κB–p65 phosphorylation between *METTL23* c.A83G iPSC-RGCs and controls (Supplemental Figure 11). This might be due to masking of secreted PS2 (36) by high amounts of supplements in the culture medium or frequent medium changes (41).

## Discussion

To our knowledge, this is the first report of a mutant histone methyltransferase leading to NTG. The aberrant splicing by mutation at the end of *METTL23* exon 2 was confirmed in exon-trapping vector–transfected HEK293T cells, the NTG patient–derived iPSCs, and *Mettl23^+/G^* mouse retinas. Moreover, our findings indicate that METTL23 was expressed in RGCs and was associated with NF-κB–mediated *TNFA* and *IL1B* transcription through dimethylation of histone H3R17. Furthermore, both the novel *METTL23 c.*A83G mutation and deficiency led to RGC loss and optic nerve head axonal degeneration in *Mettl23*-KI and -KO mice, similar to our findings in patients with NTG.

The *METTL23 c.A83G* mutation was heterozygous in all of our patients with NTG. This allele was transmitted vertically from the affected parents to the affected children, clearly indicating an autosomal dominant inheritance pattern. A second *METTL23* variant also suspected of affecting splicing (c.84+60delAT) was found in 14 individuals, reported to be unrelated, from a collection of 1,029 Japanese NTG cases and in 8 of 1,402 age-matched Japanese controls (Table 2, Figure 2B, and Supplemental Tables 5 and 6). No TBK1 copy number variations or OPTN E50K mutations were detected in any of the patients with NTG who had the *METTL23* c.84+60delAT variant (Supplemental Figure 5, A and B). The distribution of the allele between the patients and controls suggests a potential contribution to NTG (*P* = 0.03, Fisher’s exact test or χ^2^ with Yates correction; *P* = 0.038, logistic regression adjusting for age and sex, Supplemental Figure 12), however, further work, including analyses of other data sets, will be required to confirm this finding.

To date, only 2 genes, *OPTN* and *TBK1*, have been associated with familial NTG. OPTN and TBK1 proteins have essential roles in NF-κB signaling (42, 43) and autophagy (1, 2), which share common upstream signals and regulators and can regulate each other through positive or negative feedback loops (44, 45). Our data indicate that only the long isoform METTL23 catalyzed the dimethylation of H3R17 and upregulated *SP2* and *sP2* transcription in vitro and in vivo and *TNFA* and *IL1B* in vivo. TNF-α and IL-1β are particularly important for RGCs and optic nerve fibers (46–48). Loss of sP2 is associated with NF-κB–mediated activation of TNF-α and IL-1β in vivo (36). Taken together, our findings suggest that, similarly to OPTN and TBK1, METTL23 also exerts negative regulation of NF-κB–mediated TNF-α and IL-1β expression. These results suggest that NF-κB signaling proteins may also contribute to NTG and that regulation of NF-κB signaling may be a therapeutic option.

The role of histone arginine methylation in the pathogenesis of neuronal diseases is being rapidly elucidated; however, to our knowledge, this is the first report of aberrant histone arginine methylation in NTG. *OPTN* and *TBK1* mutations have also been identified in patients with amyotrophic lateral sclerosis (ALS). Another ALS-associated protein, ATXN2, has also been associated with primary open-angle glaucoma (49–51). Moreover, ALS-linked mutations in the RNA-binding protein fused in sarcoma (FUS) cause insoluble intracytoplasmic protein aggregates (25). This process was alleviated by the depletion of PRMT1, which functions as a transcriptional coactivator by depositing dimethylarginines on histone H4R3 (52). We found that haploinsufficiency of METTL23 was sufficient to impede the dimethylation of histone H3R17, thereby inducing NTG. Several pathogenic variants of METTL23 are associated with familial intellectual disability in White, Arab, and Pakistani pedigrees (53–57), suggesting etiologic links between arginine methylation, nervous system disorders, and glaucoma.

In conclusion, we have identified a *METTL23 c.A83G* mutation in patients with NTG that follows a dominant inheritance pattern and an intron 2 mutation causing aberrant splicing in unrelated NTG patients. The *Mettl2*3 c.A83G–KI murine model successfully recapitulated the clinical phenotype of patients with NTG. NTG is an irreversible blinding disease, and the diagnosis is more complex than that of high-pressure forms of glaucoma. This study gives insight into the involvement of NF-κB–mediated inflammation by histone methylation, a new understanding of NTG, and, we believe, will stimulate the development of new diagnostic and therapeutic strategies through the control of histone methylation. As the optic nerve is part of the CNS, our findings may also have implications for the pathogenesis of other neurodegenerative diseases.

## Methods

Additional details can be found in the Supplemental Methods.

### Human study participants.

Eleven (6 affected and 5 unaffected) members of a Japanese family with dominantly inherited NTG were clinically assessed. DNA samples from unrelated patients with NTG were collected from the ophthalmology departments of Tokyo Medical Center (*n* = 167), Yamanashi University (*n* = 238), Tokyo University (*n* = 435), and Tohoku University (*n* = 542), respectively, and analyzed at the Tokyo Medical Center. For all patients, a diagnosis of NTG was made according to glaucomatous changes in the optic nerve head and peripapillary retina, corresponding to visual field defects determined using the reliable Humphrey perimeter Swedish interactive threshold algorithm (SITA) standard 30-2 or 24-2 test results and an IOP, without the use of ocular hypotensive medication, that was consistently less than 22 mmHg during the follow-up period (58). None of the patients had other ocular abnormalities or systemic conditions.

### WES data analysis.

Genomic DNA were extracted from peripheral whole blood and subjected to WES by Macrogen Japan (Japan). Briefly, the WES libraries were established from the DNA samples using the Agilent SureSelect Exome Capture Kit (Agilent Technologies) and then sequenced with a HiSeq 2500 (Illumina). WES data analysis was performed with the reference human genome (hs37d5) and focused on patients’ common missense mutations occurring at low frequency (<1%) in public databases (ExAC, gnomAD, HGVD, 4.7KJPN) and our in-house database. The remaining variants were filtered by the inheritance pattern with sibling DNA. Then *METTL23* and *CEP290* were identified as the putative disease-causing genes. Finally, mathematical predictions of PolyPhen2, SIFT, and PROVEAN revealed that *METTL23* was a candidate gene with the heterozygous mutation p.E28G (c.A83G) (RefSeq ID: NM_001080510). Read depth data for each of the *METTL23* exons were extracted from the patients’ exons and then compared with the average read depth. There were no additional exon-spanning duplications or deletions in any of the patients. Sanger sequencing was performed to detect the *METTL23* c.A83G mutation using the standard method. The primer sequences are listed in Supplemental Table 7.

### Bioinformatics.

The subfamily-specific conservation at the variant’s position and neighboring amino acids were analyzed by Jalview. It is highly conserved among humans, chimpanzees, sheep, mice, cats, cattle, rats, pigs, horses, chickens, frogs, and zebrafish. To analyze the effect of the novel variant on protein structure, a prediction of the protein’s secondary structure was performed with Jpred 4. A structural model of the METTL23 protein was generated by one-to-one threading using the Phyre server (59). DNASTAR was used to visualize protein structures. As the mutation is at the end of exon 2, we used HSF version 3.0 (http://www.umd.be/HSF3/HSF.html) to analyze whether the variant could cause changes to the splice sites. HSF utilizes 2 algorithms that are position weight matrices (HSF Matrices) and maximum entropy (MaxEnt) to predict a mutation’s effect on splicing motifs including donor splice sites. We used the gene name *METTL23* and the mutant c.83A>G or c.84+60_61delAT as input and selected the longest transcript for the analysis. The c.83A>G and c.84+60_61delAT variants were predicted to result in “WT site broken” and “site broken,” respectively, which means that the variants likely affect splicing (Table 2).

### In vitro splicing assay.

PrimeSTAR HS DNA polymerase (TaKaRa) was used to amplify *METTL23* exon 2 from the affected individuals and control participants. PCR products were inserted into the pSpliceExpress vector (Addgene no. 32485) (60) using XhoI and Xbal endonucleases (TaKaRa), and the resulting constructs were verified by Sanger sequencing. WT and mutant (c.A83G) minigenes were transfected into HEK293T (American Type Culture Collection [ATCC]) cells using the ViaFect transfection reagent (Promega). Forty-eight hours later, total RNA was isolated using the RNeasy Plus Mini Kit (QIAGEN). The High-Capacity cDNA Reverse Transcription Kit (Applied Biosystems) was used with random primers to generate cDNA. The splicing vector–transcribed cDNA species were specifically amplified by PCR using the primer pairs InsEX2_F and InsEX3_R (Supplemental Table 7). The RT-PCR products from the WT and mutant minigene were visualized by gel electrophoresis, and their sequences were determined by Sanger sequencing.

### Preparation and maintenance of human iPSCs.

Human iPSCs were obtained and cultured as previously described (61). Briefly, after culturing for 5 days with plate-bound anti-CD3 mAb (BD Pharmingen) in GT-T502 medium (Kojin Bio) that contained recombinant interleukin 2 (IL-2), activated PBMCs were collected and transferred to a new 6-well plate coated with the anti-CD3 mAb. Twenty-four hours later, the SeV vector carrying OCT3/4, SOX2, KLF4, and c-MYC (Dnavec) was added. Forty-eight hours later, the infected cells were transferred to a 10 cm dish that contained mitomycin C–inactivated mouse embryonic fibroblast (MEF) feeder cells. After an additional 24 hours of incubation, the generated iPSCs were maintained on irradiated MEF feeder cells. Then, the iPSCs were transferred to feederless culture. Human iPSCs (4.5 × 10^5^) were expanded in COAT-1–coated (Cellartis def-cs 500, TaKaRa) 6-well plates in basal medium (Cellartis def-cs 500, TaKaRa).

### Differentiation and purification of iPSC-RGCs.

iPSCs were differentiated into retinal progenitor cells (RPCs) and RGCs, as described previously (41). In brief, RGC differentiation entailed the processing of uniform RPC cultures (up to day 21), RGC differentiation (days 22–36), and RGC maturation (days 37–43). On day 0, basal medium (Cellartis def-cs 500, TaKaRa) was replaced with RPC induction medium (DMEM/F12 [50:50], 1% penicillin/streptomycin, 1% Glutamine MAX (GlutaMAX Supplement, Thermo Fisher Scientific), 1% nonessential amino acid [NEAA], 0.1 mM 2-mercaptoethanol [2-ME], 2% B27 supplement, 1% N2 supplement) containing 2 μM XAV939, 10 μM SB431542, 100 nM LDN193189, 10 mM nicotinamide, and 10 ng/mL IGF1, and changed daily. On day 4, the culture medium was exchanged daily for 18 days with RPC induction medium containing 2 μM XAV939, 10 μM SB431542, 100 nM LDN193189, 10 ng/mL IGF1, and 10 ng/mL basic FGF (bFGF). Before RGC differentiation, the medium was changed using RGC induction medium containing sonic hedgehog (SHH) protein (250 ng/mL) and FGF8 (100 ng/mL) daily for 2 days. On day 24, cells were expanded into small clusters using a cross-hatching technique with Leibovitz’s medium, and then replated in RGC induction medium containing Follistatin 300 (100 ng/mL), cyclopamine (0.5 μM), *N*-[(3,5-difluorophenyl)acetyl]-l-alanyl-2-phenylglycine-1,1-dimethylethyl ester (DAPT) (3 μM), and 4.2 μM Y27632. The medium was exchanged daily for 2 days with RGC induction media containing Follistatin 300 (100 ng/mL) and DAPT (3 μM). From day 27, the medium was exchanged every 2 days with RGC induction medium containing DAPT (3 μM), Y27632 (10 μM), forskolin (5 μM), cAMP (400 μM), brain-derived neurotrophic factor (BDNF) (40 ng/mL), NT4 (5 ng/mL), and ciliary neurotrophic factor (CNTF) (10 ng/mL). After maturation (day 36), the medium was exchanged every 3 days with RGC induction medium containing DAPT (3 μM) and Y27632 (10 μM). RGCs expressing the Thy1.2 surface receptor were positively selected using the MiniMACS Separator and Starting Kit (130-090-312, Miltenyi Biotec) with CD90.2 Microbeads (130-121-278, Miltenyi Biotec). The purified cells were analyzed by immunofluorescence with anti-Brn3a antibody (1:500; AB5945, MilliporeSigma), anti-TUBB3 antibody (1:500; MMS4359, BioLegend), anti-METTL23 antibody (1:500; Thermo Fisher Scientific; PA5-71814), anti-H3R17me2 antibody (1:100; MilliporeSigma; 07-214), and anti–NF-κB–p65 (Ser536) antibody (1:100; no. 3033, Cell Signaling Technology).

### TA cloning.

Total RNA from murine retinas or iPSCs was purified using the RNeasy Mini Kit (QIAGEN) according to the manufacturer’s instructions. After cDNA synthesis using the High-Capacity cDNA Reverse Transcription Kit (Applied BioSystems), the primer pairs mMETTL23_F1/hMETTL23_F2 and mMETTL23_R1/hMETTL23_R2 were used to amplify the *Mettl23* and *METTL23* fragments, respectively. The amplified fragments were ligated into the pCR2.1 vector with the TOPO TA Cloning Kit (Invitrogen, Thermo Fisher Scientific) following Sanger sequencing. The TA cloning primer sequences are listed in Supplemental Table 7.

### Cloning of METTL23-expressing plasmids.

Full-length human *METTL23* cDNA was cloned into the pEF-BOS-FLAG vector (20) using the standard procedure. To introduce mutations into expressing vectors, splicing deletion products were obtained using the KOD Mutagenesis Kit (TOYOBO, Japan) and specific deletion primers (Supplemental Table 7) and were then validated by Sanger sequencing.

### WB analysis.

Plasmids (2 μg) containing WT METTL23-FLAG, splicing 1–FLAG (skip exon 2), and splicing 2–FLAG (skip exons 2 and 3) were transfected into subconfluent HEK293T, COS-7 (ATCC), and 661W cells (62) in a 6-well plate using ViaFect transfection reagent (Promega), respectively. Forty-eight hours later, the cells were harvested by RIPA buffer with protease and phosphatase inhibitors (Roche), PMSF, and aprotinin. Equal amounts of samples were subjected to an Any kD Mini-PROTEAN TGX Gel and transferred onto a PVDF membrane using the Transblot Turbo system (Bio-Rad). Blots were probed with a Can Get Signal and PVDF Blocking Reagent Set (TOYOBO). Every blot was also probed with an anti-actin antibody to ensure equal protein loading. Protein detection was achieved with SuperSignal West Femto Maximum Sensitivity Substrate (Thermo Fisher Scientific) using the Bio-Rad system (ChemiDoc XRS+).

### Primary antibodies.

The primary antibodies used in this study included: anti-METTL23 antibody (1:1,000; PA5-71814, Thermo Fisher Scientific); anti-H3R17me2 antibody (1:1,000; 07-214, MilliporeSigma); anti-actin antibody (1:4,000; MAB1501, MilliporeSigma), and anti-FLAG antibody (1:1,000; 9A3, Cell Signaling Technology).

### METTL23 intracellular localization assay.

Cells were transfected with either WT or variant METTL23-FLAG vectors using ViaFect transfection reagent (Promega) on 24-well coverslips (MS-92132, Sumitomo Bakelite) in 24-well plates. After fixation with 4% (PFA) and permeabilization with 0.3% Triton X-100 in PBS for 10 minutes, the transfected cells were blocked with protein block serum-free (Dako) for 1 hour and incubated with the primary antibody, anti-METTL23 antibody (1:500; Thermo Fisher Scientific; PA5-71814), and anti-FLAG antibody (1:500; CST; 9A3) overnight at 4°C. Then, Alexa Fluor 568–conjugated goat anti–mouse IgG antibodies (1:500; Invitrogen, Thermo Fisher Scientific), Alexa Fluor 488–conjugated goat anti–rabbit IgG antibodies (1:500; Invitrogen, Thermo Fisher Scientific), and DAPI (1:500; Dojindo) were used to detect FLAG, METTL23, and DNA signals, respectively. The cells were mounted with Ultramount Aqueous Permanent Mounting Medium (DakoCytomation) and visualized under a confocal fluorescence microscope (LSM700; Zeiss).

### Generation of Mettl23-KI and -KO mice.

*Mettl23*-KI and -KO mice were generated by the CRISPR/Cas9 system as previously described with minor modifications (63). In brief, designed guide RNA and tracer RNA were synthesized independently (Fasmac Ltd.). The single-stranded donor oligonucleotide (ssODN) of mouse *Mettl23* with the c.A272G to mutation that evokes an amino acid substitution identical to that of the glaucomatous c.GAG>GGC mutation in human *METTL23* was separately synthesized (Integrated DNA Technologies [IDT]). We also added c.G273C for genotyping convenience to produce a BstPI cleavage site. The mixture of Cas9 Nuclease Protein NLS (NIPPON GENE), METTL23crRNA-tracrRNA complex, and the ssODN was electroporated into C57BL/6J mouse embryos using the technique for animal knockout system by electroporation (TAKE). For genotyping of the offspring, tail specimens were collected from the pups 3 weeks after birth, and then genomic DNA was extracted and purified. Prepared genomic DNA samples were amplified by PCR using PrimeSTAR HS DNA Polymerase (TaKaRa) and directly sequenced (BigDy Terminator, version 3.1, Cycle Sequencing Kit, Thermo Fisher Scientific) on a DNA sequencer (ABI 3130, Applied Biosystems). The primers used for generation are listed in Supplemental Table 7.

Subsequent to mutation identification, restriction fragment length polymorphism (RFLP) was performed. The 500 bp region was amplified using METTL23.Geno.Seq.Fwd and METTL23.Geno.Seq.Rvs primers (Supplemental Table 7). Amplification was performed as follows: 98°C for 3 minutes, then 30 cycles at 98°C for 10 seconds, 55°C for 30 seconds, and 72°C for 30 seconds, followed by 72°C for 5 minutes. PCR products were digested with 1 unit of EcoRI (TaKaRa) and visualized on a 2% agarose gel stained with ethidium bromide. Similarly, the *Mettl23^–/–^* mutation (A74Dfs*16, A74 is substituted into D and frameshifted 16 more amino acids, and then the stop codon comes to the terminated translation) created a BsII restriction site, and PCR products were digested with BsII (TaKaRa). The *Mettl23*-KI and -KO mice were backcrossed 10 times with the C57BL/6J strain (CLEA Japan). The *Mettl23*-KI and -KO and littermate control mice were housed together in a standard animal maintenance facility under a 12-hour light/12-hour dark cycle and were regularly screened for pathogens.

### IOP measurement.

IOP was measured using an Icare TONOLAB Tonometer (iCare) approximately 2 minutes after i.p. injection of a combination anesthetic (0.3 mg/kg medetomidine, 4.0 mg/kg midazolam, and 5.0 mg/kg butorphanol) at 2, 4, and 6 months of age. IOP measurements of each eye were performed between 11 am and 2 pm to minimize the effect of diurnal IOP variation and were averaged from 4 sets of 6 recordings. Littermate controls were used for all live-animal studies.

### OCT observation.

Live imaging of retinal sections was performed using OCT on 2- to 6-month-old *Mettl23*-KI and -KI mice under a combination anesthetic (0.3 mg/kg medetomidine, 4.0 mg/kg midazolam, and 5.0 mg/kg butorphanol) injected i.p., as described previously (64). Pupils were dilated with Mydrin-P ophthalmic solution (1319810Q1053, Santen Pharmaceutical). Fundus examinations and OCT studies were performed using a Micron IV (Phoenix Technology Group). Structural analysis of OCT data was performed using INSIGHT software (Phoenix Technology Group).

### ERG.

Electroretinograms were recorded as described previously with modification (29). Briefly, mice were dark adapted overnight and anesthetized with a combination anesthetic (0.3 mg/kg medetomidine, 4.0 mg/kg midazolam, and 5.0 mg/kg butorphanol) by i.p. injection. Pupils were dilated with Mydrin-P ophthalmic solution (1319810Q1053, Santen Pharmaceutical) under dim red light, and 0.1% hyalein ophthalmic solution (1319720Q3078, Santen Pharmaceutical) was used to moisten the cornea. Gold electrodes were placed on corneal surfaces, the reference electrode was placed intraorally, and the ground electrode was inserted intra-anally. Body temperature was maintained at approximately 37°C with a heating pad. STR recordings were performed simultaneously in both eyes at an intensity range of –6 to –3.5 log scot.cd s/m^2^ using a Ganzfeld bowl (LS-100, Mayo). The responses were band-pass filtered from 0.3–50 Hz and averaged over 30 trials (PuREC PC-100, Mayo). The amplitudes of positive STRs were measured from baseline.

### Histology and immunofluorescence.

H&E staining was performed to identify the histology of *Mettl23*-KI and -KO mice. After fixing with formalin–acetic acid–alcohol (FAA) solution at 4°C overnight, the eyes were processed for general paraffin embedment as described before (64). After deparaffinization and rehydration, paraffin sections (5 μm thick) were stained with H&E. The images were collected using a Nikon Eclipse light microscope.

To identify METTL23 distribution in the monkey retinas, immunofluorescence on paraffin sections was performed. The eyes of an adult cynomolgus macaque monkey (*Macaca fascicularis*) were fixed in FAA solution at 4°C overnight. The paraffin sections prepared as described above were treated with Target Retrieval Solution (DakoCytomation) at 120°C for 15 minutes. After blocking, they were incubated with anti-METTL23 antibody (1:200; PA5-71814, Thermo Fisher Scientific) overnight at 4°C. Slides were washed 3 times with PBS and then incubated with Alexa Fluor 488–conjugated goat anti–rabbit IgG (1:500; Life Technologies, Thermo Fisher Scientific) and DAPI (1:500; Dojindo). Sections were mounted with Ultramount Aqueous Permanent Mounting Medium (DakoCytomation) and observed with a confocal fluorescence laser microscope (LSM 700, Zeiss).

To identify the role of METTL23 in NF-κB signaling, an avidin-biotin immunoperoxidase assay was performed after pretreatment as described above. Rabbit anti–p–NF-κB–p65 (Ser536) antibody (1:200; no. 3033, Cell Signaling Technology) was applied overnight at 4°C. Images were collected using a Nikon Eclipse light microscope.

For immunofluorescence of frozen section, the eyes from *Mettl23^G/G^* and *Mettl*23*^–/–^* mice were fixed with 4% PFA for 2 hours at 4°C. The tissue was processed for general cryopreservation in 10%, 20%, and 30% sucrose in PBS and then embedded in OCT compound. After cryosectioning at 15 μm thickness, the specimens were washed twice with PBS and permeabilized in 0.3% Triton X-100 with PBS for 5 minutes. After blocking, the specimens were incubated with anti-METTL23 antibody (1:50; PA5-71814, Thermo Fisher Scientific) overnight at 4°C and then with Alexa Fluor 488–conjugated rabbit anti–rabbit IgG (1:500; Life Technologies, Thermo Fisher Scientific) and DAPI (1:500; Dojindo). Slides were sealed with coverslips using Ultramount Aqueous Permanent Mounting Medium (DakoCytomation) and analyzed with a LSM 700 (Zeiss).

### Retinal flat-mount and RGC counts.

Eyes were dissected and immunostained in flat mounts as described previously (20). Briefly, dissected eyes were fixed in 4% PFA overnight at 4°C and carefully dissected. Nonspecific binding was prevented by blocking with Dako’s serum-free blocking buffer, and all specimens were incubated with anti-Brn3a antibody (1:200; AB5945, MilliporeSigma) at 4°C for 5 nights. After incubation with Alexa Fluor 568–conjugated rabbit anti–rabbit IgG (1:1,000; Life Technologies, Thermo Fisher Scientific), retinas were mounted in Ultramount Aqueous Permanent Mounting Medium (DakoCytomation). Four micrographs (700 × 700 μm) per single retinal specimen from the mid-peripheral region of quadrants 1.5–2.0 mm from the optic head were imaged using an LSM 700 confocal fluorescence microscope (Zeiss). Brn3a^+^ RGCs were counted using ImageJ (NIH) and averaged for at least 3 retinas per group.

### Quantification of optic nerve axons.

Optic nerve cross-sections were prepared and imaged as described previously (31). In brief, eyes (globes) were enucleated along with the retrobulbar optic nerves. After fixation with 4% PFA overnight at 4°C, the retrobulbar optic nerves were dissected approximately 1 mm distal to the globe and postfixed in PBS with 2.5% glutaraldehyde and 10% formalin for 24 hours. After incubation in 1% osmium for 2 hours and dehydration, nerves were embedded in Technovit 7100 (RT7100, Kulzer). Cross-sections (1.5 μm thick) were stained with 1% paraphenylenediamine in absolute methanol for 15 minutes. Axon counting was performed in 20 nonoverlapping representative areas of each nerve cross-section at ×100 magnification (Nikon) and measured in 5 cross-sections per optic nerve using AxonJ (NIH).

### Purification of METTL23-his6-FLAG protein.

HEK293T cells were transfected with pEF-BOS-METTL23-his6-FLAG, pEF-BOS-Splicing1-his6-FLAG, pEF-BOS-Splicing2-his6-FLAG, and empty vectors, respectively. Forty-eight hours later, total protein was extracted with His-Lysis buffer (20 mM Tris-HCl, pH 8.0, 500 mM NaCl, 0.5% NP-40, 1 mM PMSF, and protease/phosphatase inhibitor [Roche]). After centrifugation at 18,900*g* for 10 minutes, the supernatants were incubated with Ni-NTA Agarose (QIAGEN) for 2 hours at 4°C with gentle rotation. The Ni-NTA beads were washed 3 times with His-Lysis buffer and an additional 3 times with wash buffer (50 mM Tris-HCl, pH 8.0, 20 mM imidazole, and 0.1% NP-40). Then, they were eluted with 500 μL elution buffer (50 mM Tris-HCl, pH 8.0, 250 mM imidazole, and 0.1% NP-40) and diluted with 1.5 mL His-Lysis buffer. Next, anti-FLAG affinity gel (MilliporeSigma) was added to the diluted eluates and incubated for 2 hours at 4°C with gentle rotation. Finally, the FLAG M2 beads were washed twice with His-Lysis buffer and once with 50 mM Tris-HCl at pH 8.0.

### In vitro methylation assay.

Recombinant histone H3.1 protein (1.5 μm) (M2503S, New England BioLabs) was incubated in reaction buffer (50 mM Tris-HCl, pH 8.5, 5 mM DTT) with METTL23-immobilized FLAG M2 agarose beads (A2220, MilliporeSigma) and 15 nM SAM (ab142221, Abcam) for 3 hours at 30°C. The reaction was stopped by adding 4× Laemmli SDS Sample Buffer (Bio-Rad). Next, the methylated products were detected by WB with anti-H3R17me2 antibody (1:1,000; 07-214, MilliporeSigma).

### RT-qPCR.

Total RNA was extracted from transfected cells, iPSCs, and mouse tissue using the RNeasy Plus Mini Kit (QIAGEN) according to the manufacturer’s instructions. The yield was determined with a NanoDrop ND-1000 spectrophotometer (NanoDrop Technologies). RT-qPCR was performed with synthesized cDNA using SsoAdvanced Universal SYBR Green Supermix (Bio-Rad) using the ABI STEP-One Real-time PCR system (Life Technologies, Thermo Fisher Scientific). GAPDH was used as the normalizing control, and the primer sequences for RT-qPCR are listed in Supplemental Table 7.

### Statistics.

An unpaired, 2-tailed Student’s *t* test or 1-way ANOVA followed by Tukey’s multiple-comparison test was used for statistical analyses via GraphPad Prism 9 (GraphPad Software). All data are presented as the mean ± SEM. *P* values of less than 0.05 were considered statistically significant.

### Study approval.

Our human studies adhered to the Declaration of Helsinki’s tenets and were compliant with the Health Insurance Portability and Accountability Act. This study was approved by the ethics committee of the Tokyo Medical Center (no. R20-2025). The animal experiments were carried out in accordance with the NIH’s *Guide for the Care and Use of Laboratory Animals* (National Academies Press, 2011) and the Association for Research in Vision and Ophthalmology Statement for the Use of Animals in Vision Research and were approved by the Committee for Animal Experiments of the National Hospital Organization Tokyo Medical Center (no. 19-DO-01).

## Author contributions

TI and YP conceived and designed the study, analyzed the data, and wrote the initial draft of the manuscript. YP, AS, YM, MN, DI, NM, and MY performed the laboratory work. IK, CK, FM, KK, MT, M Araie, YS, TN, and M Aihara collected the samples. KY performed the data analysis. All authors reviewed and contributed to the final manuscript.

## Supplementary Material

Supplemental data

## Figures and Tables

**Table 1:**
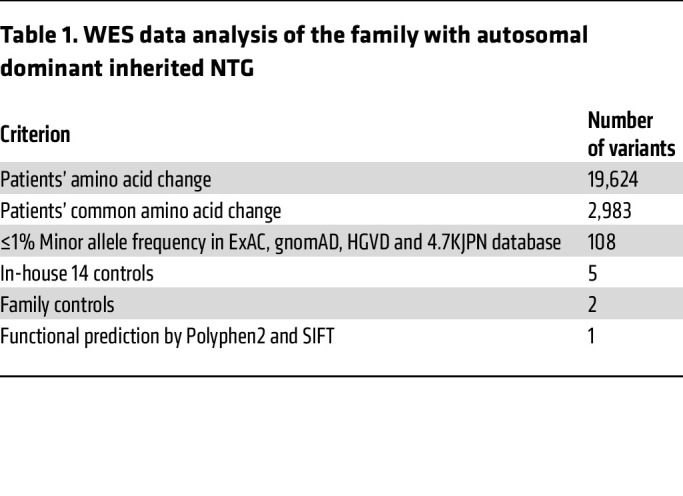
WES data analysis of the family with autosomal dominant inherited NTG

**Table 2:**
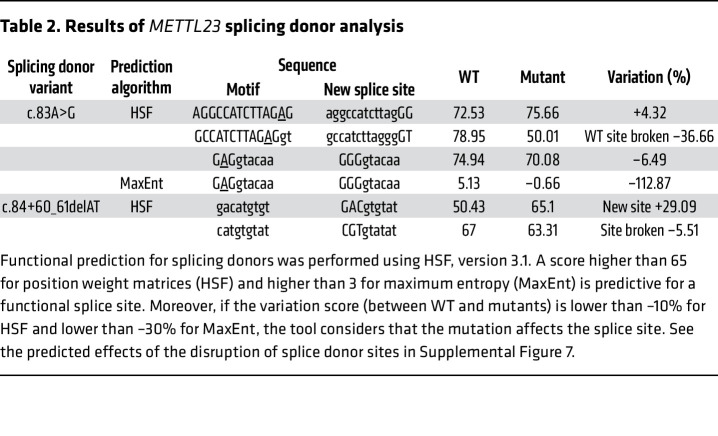
Results of *METTL23* splicing donor analysis

## Version 1 09/13/2022

In-Press Preview

## Version 2 11/01/2022

Electronic publication
