# Supplementary material for: METTL23 mutation alters histone H3R17 methylation in normal-tension glaucoma
Source: J Clin Invest. 2022 Nov 1;132(21):e153589. doi: 10.1172/JCI153589 (PMC9621137; doi:10.1172/JCI153589)
Supplement: Supplemental data [file jci-132-153589-s091.pdf]

Supplemental Material for

METTL23 mutation alters histone H3R17 methylation in normal-tension glaucoma

Yang Pan, Akiko Suga, Itaru Kimura, Chojiro Kimura, Yuriko Minegishi, Mao Nakayama, Daisuke Iejima, Kazutoshi Yoshitake, Naoko Minematsu, Megumi Yamamoto, Fumihiko Mabuchi, Mitsuko Takamoto, Yukihiro Shiga, Makoto Araie, Kenji Kashiwagi, Makoto Aihara, Toru Nakazawa, Takeshi Iwata*

*Corresponding author. E-mail: takeshi.iwata@kankakuki.jp

This PDF file includes:

Supplemental methods
Tables S1 to S7
Figures. S1 to S12
References

Supplemental methods

Validation of METTL23 antibody

The pCMV/his tagged-Mettl23 plasmid (RDB13316) expressing mouse Mettl23 was provided by RIKEN BRC through the National Bioresource Project of MEXT, Japan. Sub-confluent HEK293T cells were transfected in a 6-well plate using ViaFect transfection reagent (Promega). Forty-eight hours later, they were harvested by RIPA buffer with protease and phosphatase inhibitor (Roche), phenylmethylsulfonyl fluoride, and aprotinin. Western blot was performed as above. The primary antibodies included METTL23 antibody (1:1000; ThermoFisher; PA5-71814) and His-tag antibody (1:500; sc-8036; Santa Cruz Biotechnology).

Confirmatory of OPTN (E50K) and CNVs of TBK1

Sanger sequencing was performed to identify the OPTN (E50K) mutation, as described previously (1). Bidirectional sequencing of purified PCR products using a BigDye™ Terminator v3.1 Cycle Sequencing Kit (Thermo Fisher Scientific) was performed on an ABI 3130XL sequencer (Applied Biosystems).

Copy number variations were confirmed by qPCR assay (TaqMan, BioRad) in DNA collected from peripheral blood as described previously (2). No significant difference was detected between the amount of TBK1 PCR product produced from the DNA of NTG patients with *METTL23* c.A83G or c.84+60delAT mutation and controls (Student’s t-test).

Association analysis of *METTL23* c.84+60delAT with NTG

After DNA extraction from blood samples using the Magtration System 8Lx (Precision System Science, Japan), Sanger sequencing was performed to screen the *METTL23* c.84+60delAT variant using BigDye™ Terminator v3.1 Cycle Sequencing Kit (Thermo Fisher Scientific) on a DNA sequencer (ABI 3130; Applied Biosystems) according to the manufacturer’s instructions. The deletion (c.84+60delAT) was identified by Poly Peak Parser (http://yosttools.genetics.utah.edu/PolyPeakParser/) and was verified using the parsed file (.sqd) by DNASTAR as previously described (3). The Primer sequences were listed in Supplemental Table 7.

Student's *t*-test and χ^2 test were applied to compare the means of age and gender proportion between NTG patients (without *METTL23* c.A83G mutation) and control groups using GraphPad Prism 9, respectively. Fisher’s exact test (P=0.037, OR=2.38) and Chi-square with Yates’ correction test (P=0.036, OR=2.37) were performed to compare the difference in the minor allele frequency of *METTL23* c.84+60delAT between cases and controls by GraphPad Prism 9. Multiple logistic

regression (AICc, Tjur's R squared, Hosmer-Lemeshow goodness-of-fit test) was used to assess the simultaneous effect of *METTL23* c.84+60delAT variant, age, and gender on incident NTG by GraphPad Prism 9. A *P* value of <0.05 suggested a potential association.

Supplemental Table 1. Demographic and clinical characteristics of the NTG family.

Pedigree symbol	Diagnosis	Sex	Age at diagnosis (years)	Laterality	Max IOP, right eye (mmHg)	Max IOP, left eye (mmHg)	Cup-to-disc ratio, right eye (first examination)	Cup-to-disc ratio, left eye (first examination)	Glaucoma surgeries	Toptical treatment after diagnosis
II-1	No glaucoma	Male	-	-	-	-	0.3	0.3	None	-
II-2	NTG	Male	63	Bilateral	14	14	0.9	0.9	None	Timolol-brinzolamide combination, latanoprost, brimonizine, ripasdil
II-3	No glaucoma	Male	-	-	-	-	0.2	0.2	None	-
II-4*	NTG	Male	47	Bilateral	-	12	-	0.9	None	Timolol, brinzolamide, latanoprost, bunazosin
II-6	NTG	Female	53	Bilateral	19	18	0.8	0.8	None	Carteolol, brinzolamide, unoprostone, bunazosin
II-7	No glaucoma	Male	-	-	-	-	<0.2	<0.2	None	-
II-8	NTG	Female	47	Bilateral	14	15	0.9	0.9	None	Timolol-brinzolamide combination, latanoprost, brimonizine, bunazosin
III-5	NTG	Male	35	Bilateral	11	11	0.9	0.9	None	Timolol, travoprost, brinzolamide
III-6	No glaucoma	Male	-	-	-	-	0.2	0.2	None	-
III-7	NTG	Female	41	Bilateral	19	18	0.8	0.9	None	NA
III-10	No glaucoma	Male	-	-	-	-	0.2	0.2	None	-

*Contralateral eye is an ocular prosthesis because of birth injury.

Supplemental Table 2. Sequencing information.

Sample	Total reads produced	Average coverage (fold)	Percentage of region above 5X
II-1	114966318	108.68	97.6
II-2	109489084	93.53	96.9
II-3	148786260	146.03	98
II-4	112517828	114.52	97.5
II-6	114100546	115.95	97.4
II-7	116564460	121.33	97.5
II-8	103432842	107.13	97.2
III-5	111123700	108.06	97.6
III-6	111882626	110.07	97.7
III-7	158993120	145.47	96.6
III-10	113749634	116.88	97.8

Supplemental Table 3. In silico functional prediction of candidate variants.

Gene symbol	Variant (cDNA;protein)	RefSeq	Polyphen2_HVAR_pred	Polyphen2_HVAR_score	SIFT_pred	SIFT_score	PROVEAN_pred	PROVEAN_score
METTL23	c.A83G; p.E28G	NM_001080510	Damage	0.982	Damge	0.204	Deleterious	-6.95
CEP290	c.A66C; p.E22D	NM_025114	Benign	0.083	Tolerant	0.001	Neutral	0.04

Supplemental Table 4. Closest homologs of human METTL23 in *Homo sapiens*.

Protein	PDB	Chains	Sequence Length	Blast E-value
METTL21A	4lec	A, B	212	**3.00E-11**
METTL21D	4lg1	A, B, C	224	5.00E-07
METTL21B	4qpn	A	227	1.00E-06
METTL21C	4mtl	A, B	244	3.00E-06

Hits with e-value < 1E-5 considered to be homologs. The best hit is indicated in bold.

Supplemental Table 5. Demographic features of the study subjects (NTG patients without *METTL23* c.A83G mutation and controls).

Phenotype	Subjects	Female (%)	Mean age ±SD (years)
NTG	1029	57.92%	71.10±14.15
Control	1402	52.71%	65.25±19.88

NTG: normal-tension glaucoma.

Supplemental Table 6. Demographic and clinical characteristics of NTG patients with *METTL23* c.84+60delAT variant.

Sample ID		Diagnosis	Sex	Age at diagnosis (years)	Laterality	Max IOP, right eye (mmHg)	Max IOP, left eye (mmHg)	Cup-to-disc ratio, right eye (first examination)	Cup-to-disc ratio, left eye (first examination)	Visual field tests (MD), right eye (dB)	Visual field tests/(MD), left eye (dB)	Glaucoma surgeries
Yamanashi	Y4_A08	NTG	F	49	Bilateral	16	16	0.80	0.80	-5.96	-3.17	None
	Y4_H03	NTG	M	63	Bilateral	20	20	0.90	0.95	-29.52	-29.52	None
	Y6_F04	NTG	M	64	Unilateral	12	17	0.70	0.80	-0.64	-11.91	None
Other	GN2_12F	NTG	F	65	Bilateral	14	12	0.7	0.9	-10.47	-26.78	None
	GN3_08A	NTG	M	65	Bilateral	14	12	0.7	0.9	-4.93	-22.86	None
	GN3_10E	NTG	M	45	Unilateral	16	17	0.7	0.6	-5.09	-0.38	None
	GN3_11A	NTG	M	81	Bilateral	19	14	0.8	0.8	-5.58	-5.59	None
	GN5_12E	NTG	F	82	Bilateral	13	14	0.7	0.7	-7.19	-7.96	None
	t005	NTG	M	40	Bilateral	14.4	12.9	NA	NA	-5.2	-5.95	None
	t089	NTG	M	56	Unilateral	15.1	15.4	NA	NA	-1.69	-9.71	None
	t234	NTG	M	60	Bilateral	16.5	16.5	NA	NA	-12.67	-11.05	None
	JJ45	NTG	M	65	Unilateral	12	13	NA	NA	0.43	-8.1	None
	JJ27	NTG	F	67	Bilateral	18	18	NA	NA	-5.21	-5.42	None
	KK0805221	NTG	F	70	Bilateral	10	11	0.7	0.9	-3.87	-0.18	None

Supplemental Table 7. Information of primers related to experimental procedures.

Name	Sequence(5'to 3')	Application
hMETTL23_F1	AATGATCCCTGATACTGTGACA	Direct sequencing of exon 2 in patients
hMETTL23_R1	AGGGTTCAAGCAATTCTGTTTC	
mMettl23_F1	TGTGTTTGCCATGGACAGTG	METTL23/Mettl23 TA-cloning
mMettl23_R1	CCTAGGCATGACAGCACAGC	
hMETTL23_F2	GGGGCTGTCCTGGAGGT	
hMETTL23_R2	GTTTGTGTGCCAGGTTGCTC	
hMETTL23_splicing_XhoI_F	GTTCTCGAGCTCGTTGCCTCTCTACAGT	*In vitro* splicing assay
hMETTL23_splicing_XhoI_R	GTAGCGGCCGCAGTTCAGGGGCTTCATCTA	
hMETTL23_splicing_delAT_F	GTGTATATTGCTTGTTTTTAGCTTTTG	
hMETTL23_splicing_delAT_R	GTCAACTTAACACATCTGAACATAACC	
InsEX2_F	CAGCACCTTTGTGGTTCTCA	
InsEX3_R	AGAGCAGATGCTGGTGCAG	
M13_F	GTAAAACGACGGCCAG	TA-cloning
M13_R	CAGGAAACAGCTATGAC	
T7-Case9_F	TTAATACGACTCACTATAGGGAGAATGGACTATAAGGACCACGAC	CRISPR/Cas9 system
T7-Cas9_R	GCGAGCTCTAGGAATTCTTAC	
Mettl23.gRNA1.E91G.F	GAGTCCTGGCCCAATACCTG	
Mettl23.gRNA2.E91G.F	GCCCAGTATTTTGGTAACTC	
ssDonorOligo.for.Mettl23.E91G	GTATGGAATGTATGTTTGGCCCTGTGCTGTAGTCCTGGCCCAATAT CTCTGGTTTCACAGAAGATCTCTACCAGGCAAAGCTGTCTTAGGCG TAACCAGAATTCCCAAAATACTGGGTTATATTTGCTGTACCTTCATG CATTTAAA	
mMettl23.Geno.Seq.Fwd	ATTGTAATAAAAGGACTGGTTTCAGAACCT	Mouse genotyping
mMettl23.Geno.Seq.Rvs	AGGCAGGCAGATTTCTGAGTAAAAATACTT	
hMETTL23_cflag_splicing1_F	ATTGGAGCTGGAGTGAGCCTTCCAG	hMETTL23 plasmids
hMETTL23_cflag_splicing2_F	ATTTTGAAGACATTTTGGCTACAAT	
hMETTL23_cflag_splicing1/2_R	CATGGTGGCCGTACCTAGAGAATTCC	
hMETTL23_chis-cflag_F	CACCATCACCATCACCATGACTACAAGGACGATGACGATAAGTGAG GATCCG	
hMETTL24_chis-cflag_R	GAGACTGTCCTTTGCAAAGGAAATGACCAGC	
hMETTL23_chis/cflag_sequencing_F	CTTGGTTCATTCTCAAGCCTCAGAC	
hMETTL24_chis/cflag_sequencing_R	ATATGGTCGACGGATCCTCACTTATC	
mMyc _F	TCCTGTACCTCGTCCGATTC	RT-qPCR
mMyc _F	TCTTCAGAGTCGCTGCTGGT	
mTff1_for	CCATGACTCACCCTGCTTTT	
mTff1_rev	CCCCTACTGTGCTGAGAGATG	
mPges_F	TTA GAG GTG GGC AGG TCA GAG	
mPges_R	CCA CTC GGG CTA AGT GAG AC	
mIgfbp_F	TCGTGTGTGTGTGTTTATAG	
mIgfbp_R	ATTCCTATTGCTACACTCAG	
mEgr3_F	GCCAGGACAACATCATTAGC	
mEgr3_R	GATACATGGCCTCCACGTC	
mTNF-α_F	GCCTCTTCTCATTCCTGCTTG	
mTNF-α_R	CTGATGAGAGGGAGGCCATT	
mIl1β_F	CTTTGAAGAAGAGCCCATCC	
mIl1β_R	CATCTCGGAGCCTGTAGTGC	
pS2 -for	CCAGACAGAGACGTGTACAGTGG	
pS2 -re	ACACTGGGAGGGCGTGAC	
PTGES-for	AAGTGAGGCTGCGGAAGAAG	
PTGES-re	CCAGGAAAAGGAAGGGGTAG	
EGR3-for	CCAGAAAGGCAGGCTTCAAC	
EGR3-re	GGTGATGACAAAGGGCAAAA	
IGFBP4-for	AGCCCTCTGACAAGGACGAG	
IGFBP4-re	CGAATTTTGGCGAAGTGCTT	
MYC-for	TCTTGGCAGCAGGATAGTCCTT	
MYC-re	CGTCTCCACACATCAGCACAA	

II-2

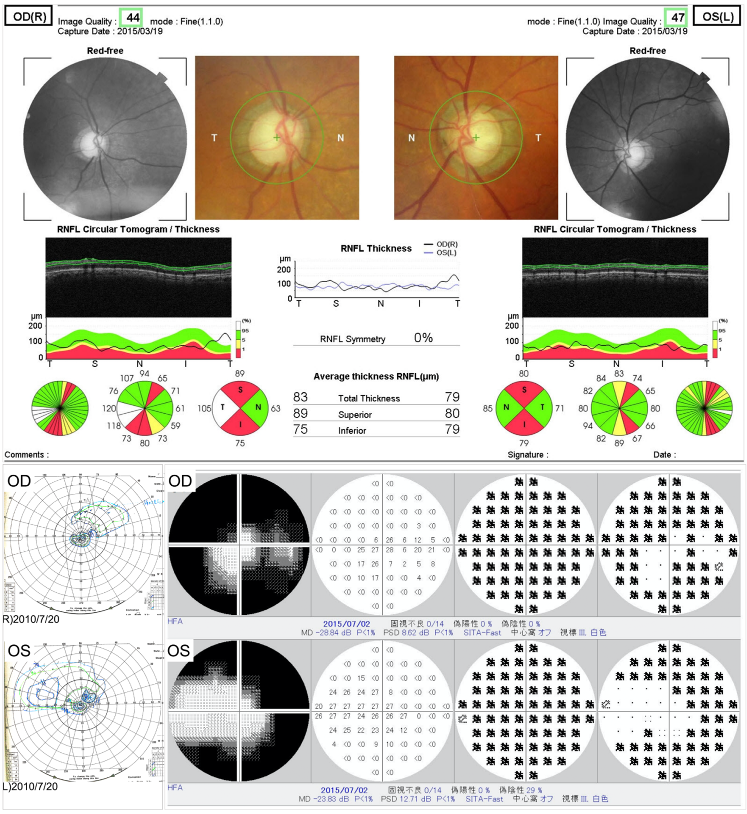
OD(R)
Image Quality : 44 mode : Fine(1.1.0)
Capture Date : 2015/03/19
mode : Fine(1.1.0) Image Quality : 47
Capture Date : 2015/03/19
OS(L)
Red-free
RNFL Circular Tomogram / Thickness
RNFL Thickness
OD(R)
OS(L)
RNFL Symmetry 0%
Average thickness RNFL(μm)
83 Total Thickness 79
89 Superior 80
75 Inferior 79
Comments :
Signature :
Date :
OD
OS
R)2010/7/20
L)2010/7/20
HFA
2015/07/02
MD -28.84 dB P<1% PSD 8.62 dB P<1% SITA-Fast
MD -23.83 dB P<1% PSD 12.71 dB P<1% SITA-Fast

II-4

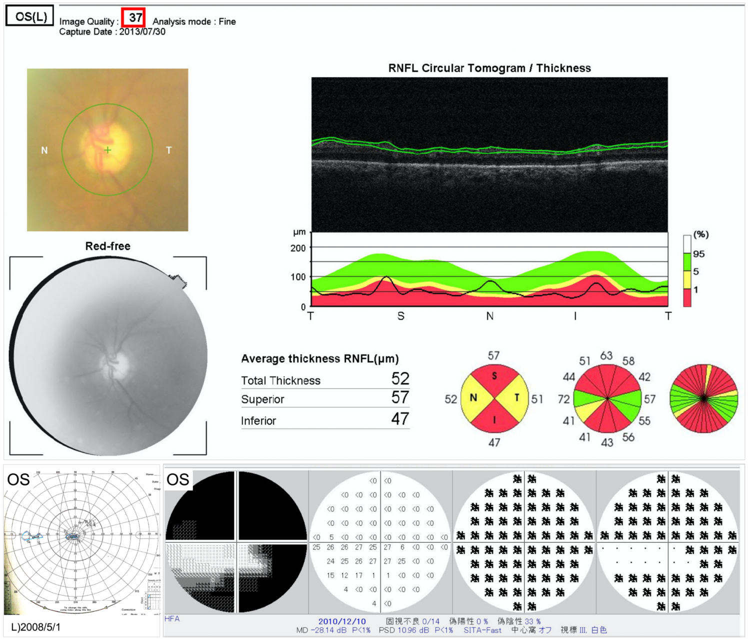
OS(L)
Image Quality : 37 Analysis mode : Fine
Capture Date : 2013/07/30
RNFL Circular Tomogram / Thickness
Red-free
Average thickness RNFL(μm)
Total Thickness 52
Superior 57
Inferior 47
OS
L)2008/5/1
HFA
2010/12/10
MD -28.14 dB P<1% PSD 10.96 dB P<1% SITA-Fast

II-6

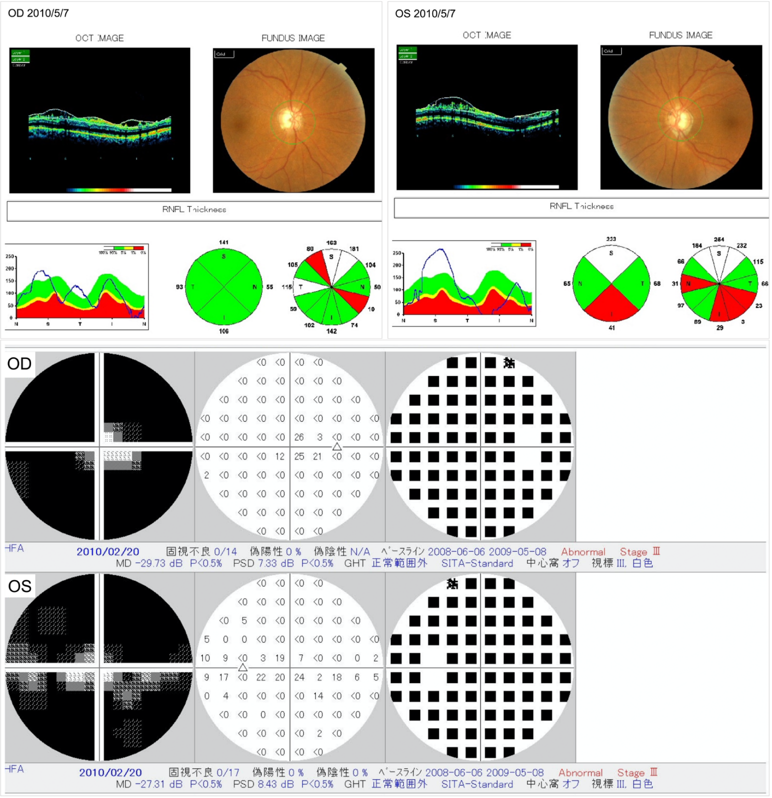

III-5

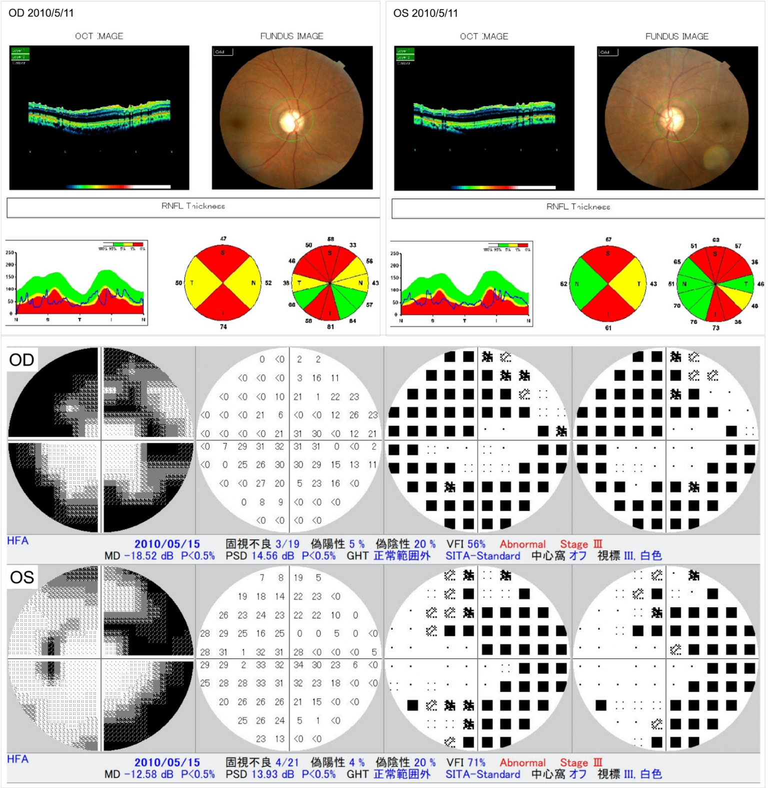

Supplemental Figure 1. Clinical manifestations of patients in the NTG pedigree. Limited information was available for Patient III-7 (Supplementary Table 1).

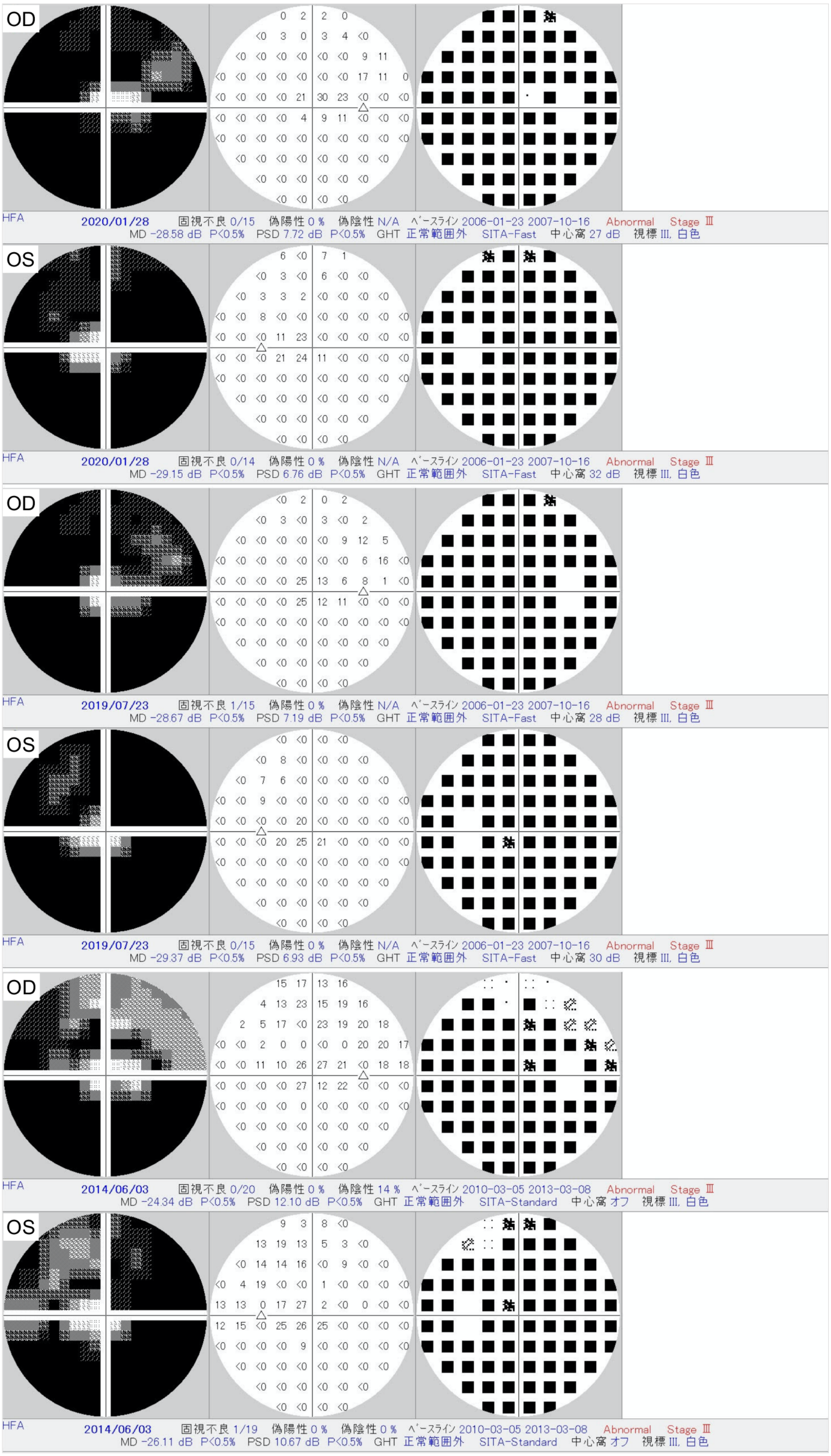

Supplemental Figure 2. Humphrey visual field results of NTG Patient II-8 (Figure1, A and B) show a progressive visual field loss.

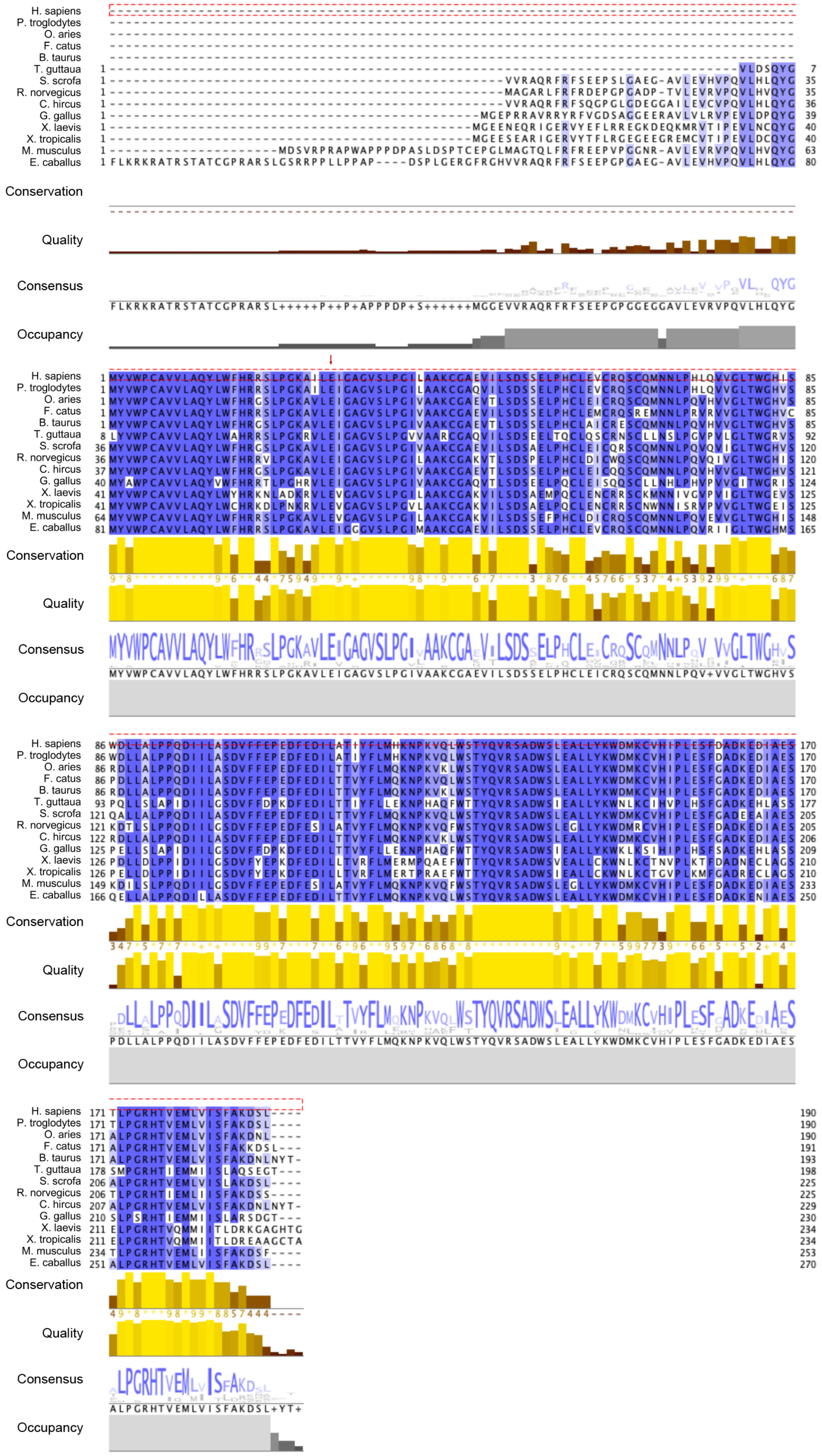

Supplemental Figure 3. Subfamily-specific conservation for METTL23. Histograms show degrees of conservation. The variant position is shown above.

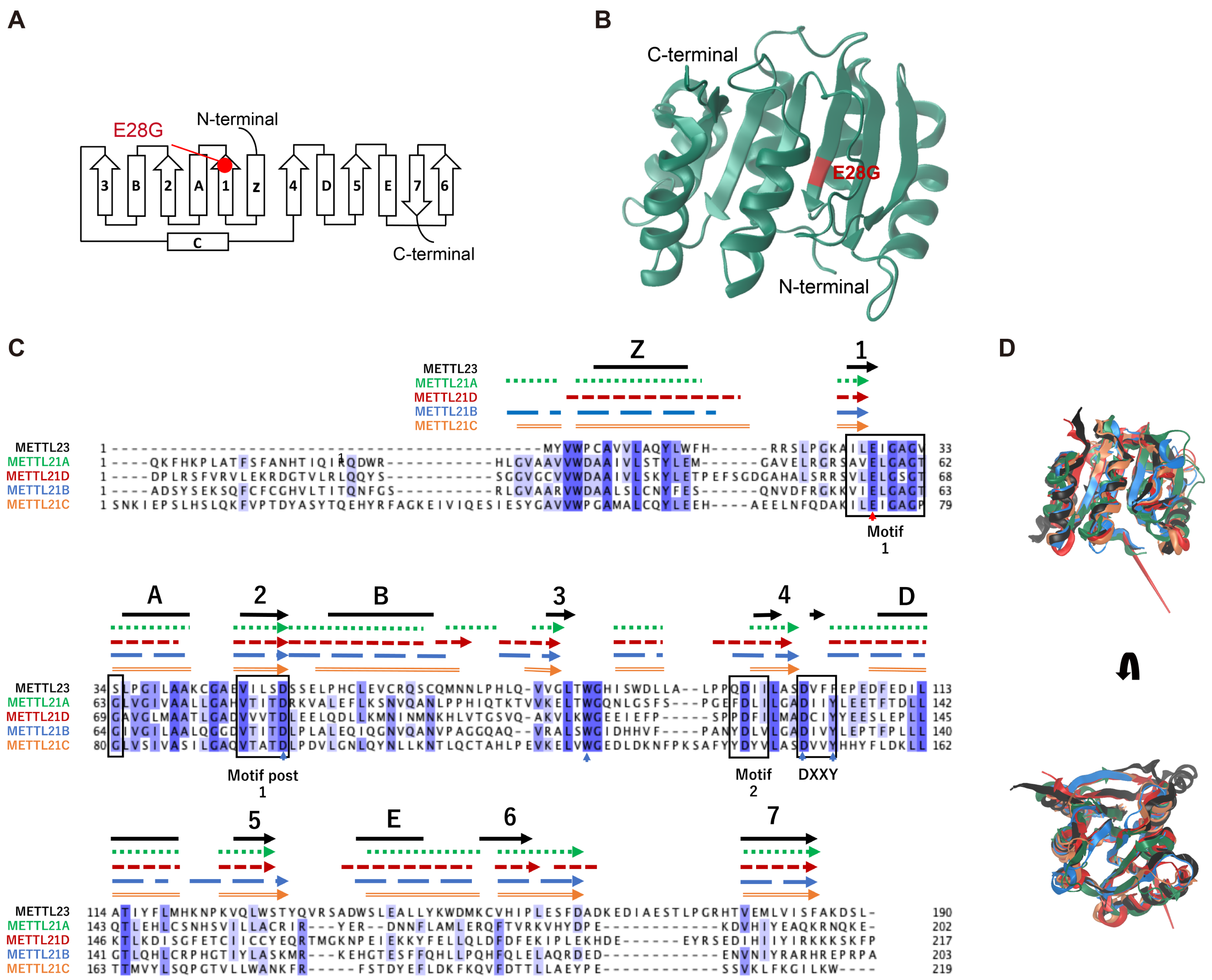

Supplemental Figure 4. Computational analysis of *METTL23* c.A83G mutation. (**A**) Topology diagram of the canonical 7BS MTase fold with the location of the identified variant. Arrows and rectangles indicate beta-strands and alpha-helices, respectively. (**B**) Predicted structural model of human METTL23 generated by one-to-one threading with phyre2, using METTL21D (c4lg1A) as a template. 176 residues (93% of METTL23) have been modeled with 100% confidence by the single highest scoring template. The location of p.E28G is marked as red. (**C**) Protein sequence alignment of human METTL23 and METTL21 proteins. Motifs 1, Post 1, Post 2, and the DXXY motif are indicated by boxes. The secondary structure prediction for METTL23 was performed with Jpred 3 (black). β-strands and α-helices are indicated by arrows and thick lines, respectively, and the numbering/lettering of these are as outlined in **A**. Red and blue arrows indicate the locations of identified variant and conserved active site residues, respectively. (**D**) Structural alignment of human METTL23 and METTL21 proteins (color code as in **C**). The alignment shows a high level of similarity.

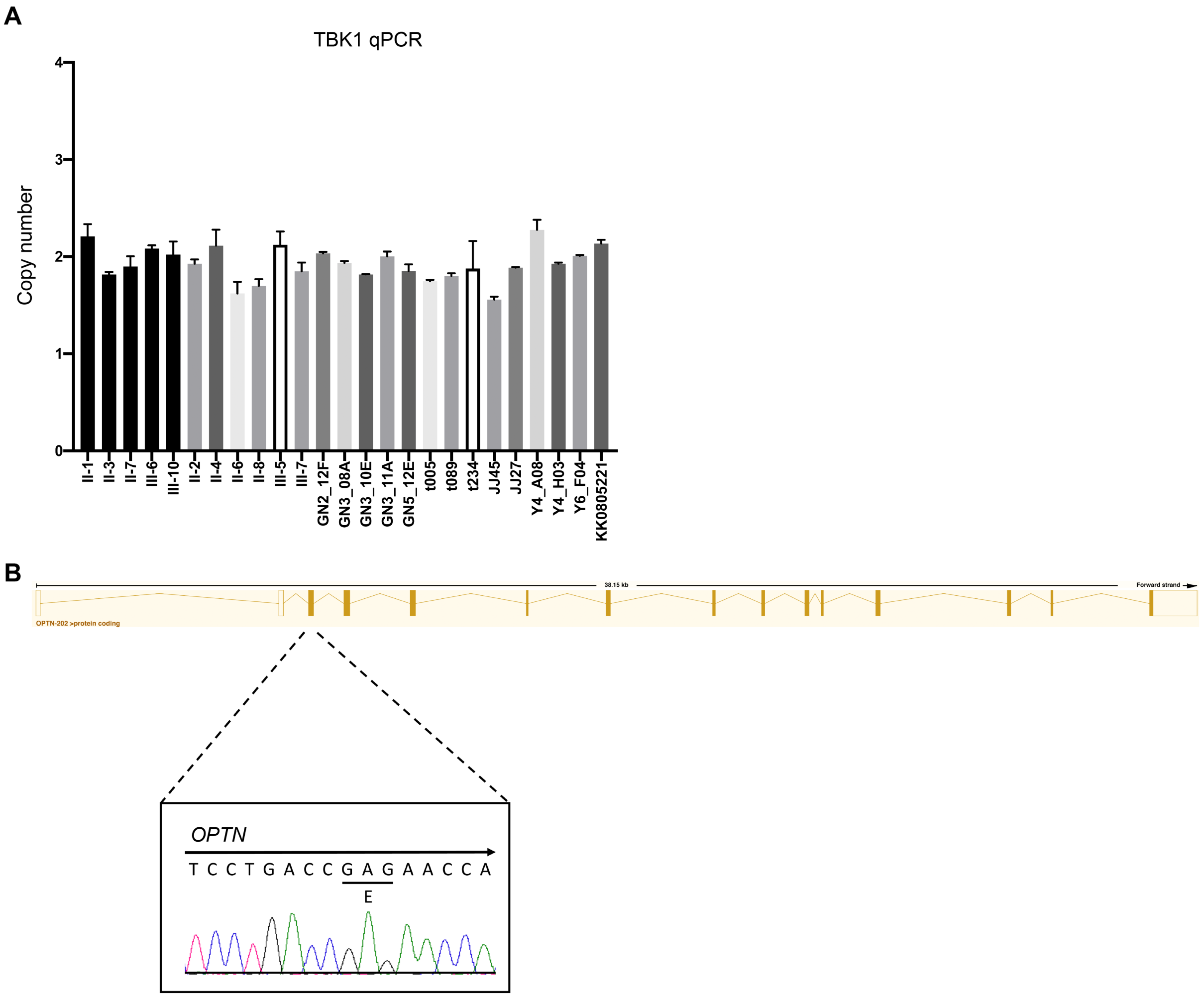

Supplemental Figure 5. Confirmation of OPTN (E50K) and CNVs of TBK1. NTG patients with *METTL23* c.A83G or c.84+60delAT variant were screened for the OPTN (E50K) mutation and *TBK1* copy number variations. (**A**) Quantitative PCR assessment of *TBK1* gene dosage. The number of copies of the *TBK1 gene* was assessed in genomic DNA collected from NTG patients with *METTL23* c.A83G or c.84+60delAT mutations and controls (II-1, II-3, II-7, III-6, and III-10). (**B**) No OPTN (E50K) missense mutation was detected by direct Sanger sequencing.

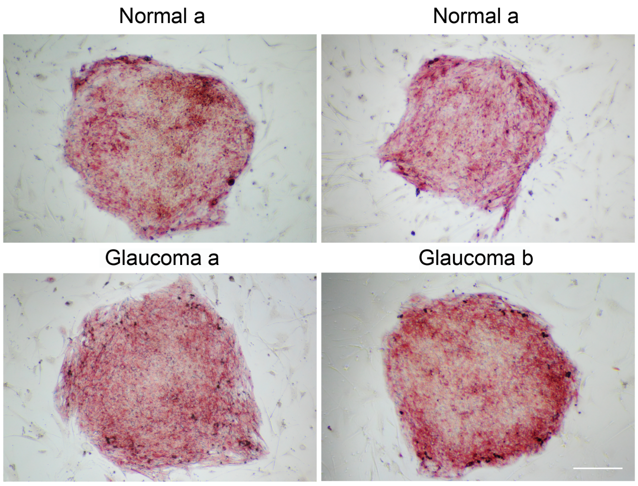

Supplemental Figure 6. Alkaline phosphatase staining for iPSCs. iPSCs were generated using peripheral blood lymphocytes obtained from two affected and two unaffected individuals, indicated by red box in Figure 1A. Scale bar: 200 µm.

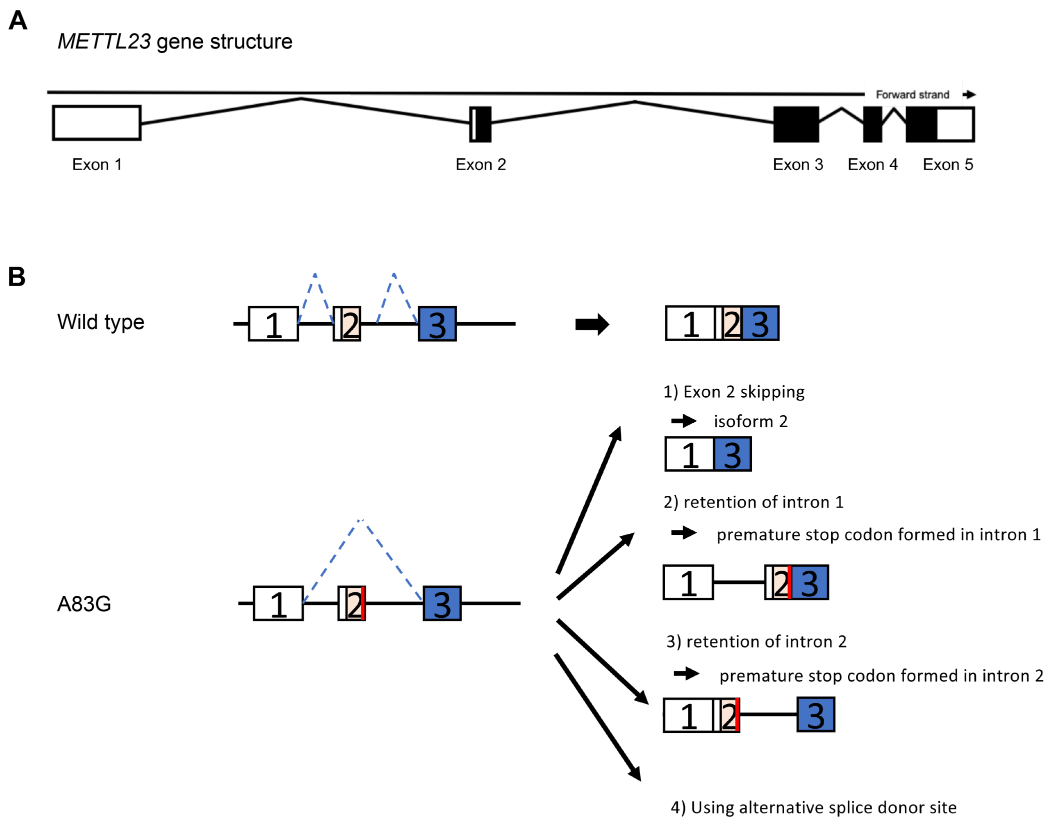

Supplemental Figure 7. Predicted effects of *METTL23* c.A83G in human retina. (**A**) *METTL23* gene structure. (**B**) Splicing predictions for the mutation in the retina of affected individuals.

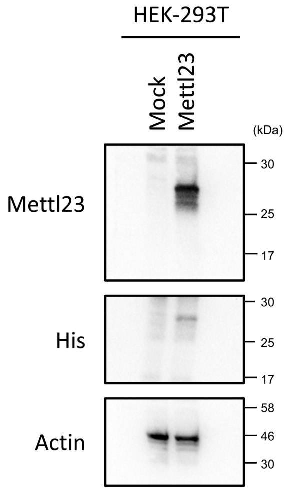

Supplemental Figure 8. Validation of METTL23 antibody. Western blot of anti-METTL23 antibody with recombinant mouse Mettl23, achieved by using pCMV/his tagged-Mettl23 plasmid.

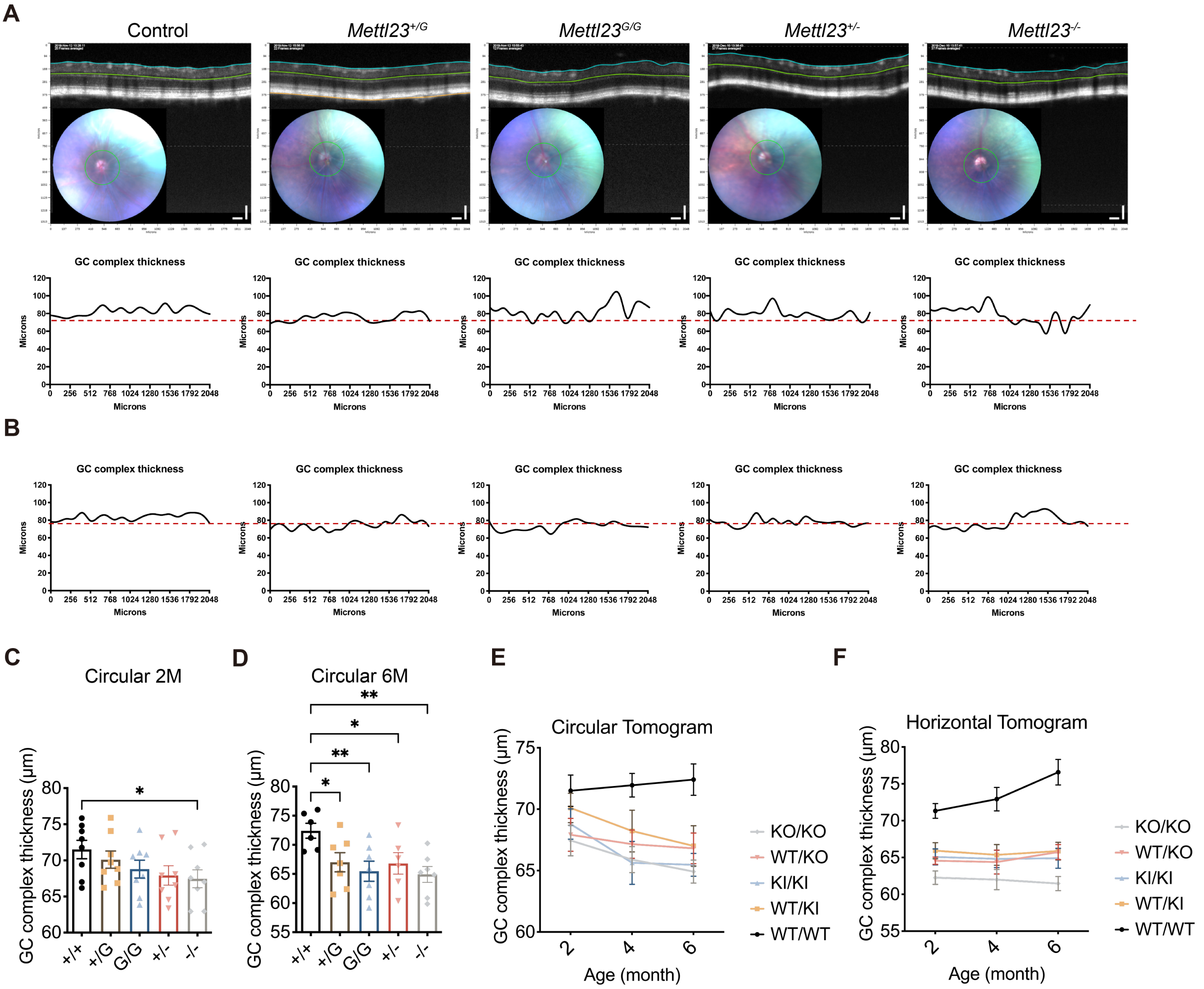

Supplemental Figure 9*. METTL23* c.A83G mutation and deficiency cause morphologic change in RGCs by OCT. Representative OCT data was obtained from control mice, *Mettl23* knock-in and knockout mice (*Mettl23*-mice) by B-circular scan (**A, B**). Peripapillary ganglion cell (GC) complex thicknesses of *Mettl23*$^{+/G}$, *Mettl23*$^{G/G}$, *Mettl23*$^{+/-}$ and *Mettl23*$^{-/-}$ mice were measured with Insight (Phoenix) and compared with *Mettl23*$^{+/+}$ at 2 months (**C**) and 6 months of age (**D**), respectively (n ≥ 6 per group). (**E**) GC complex thickness by B-circular scan in different mouse strains. (**F**) GC complex thickness by B-Horizontal scan in different mouse strains. GC complex, retinal nerve fiber layer (RNFL)/ganglion cell layer (GCL)/inner plexiform layer (IPL). Fundus images of *Mettl23*$^{G/G}$ mice in Figure **6A** (Horizontal scan) and supplemental Figure **9A** (circular scan) are obtained from the same mouse at the same time. Data are presented as mean ± SEM. ***P*<0.01, **P*<0.05 by one-way ANOVA followed by Tukey's multiple comparison test.

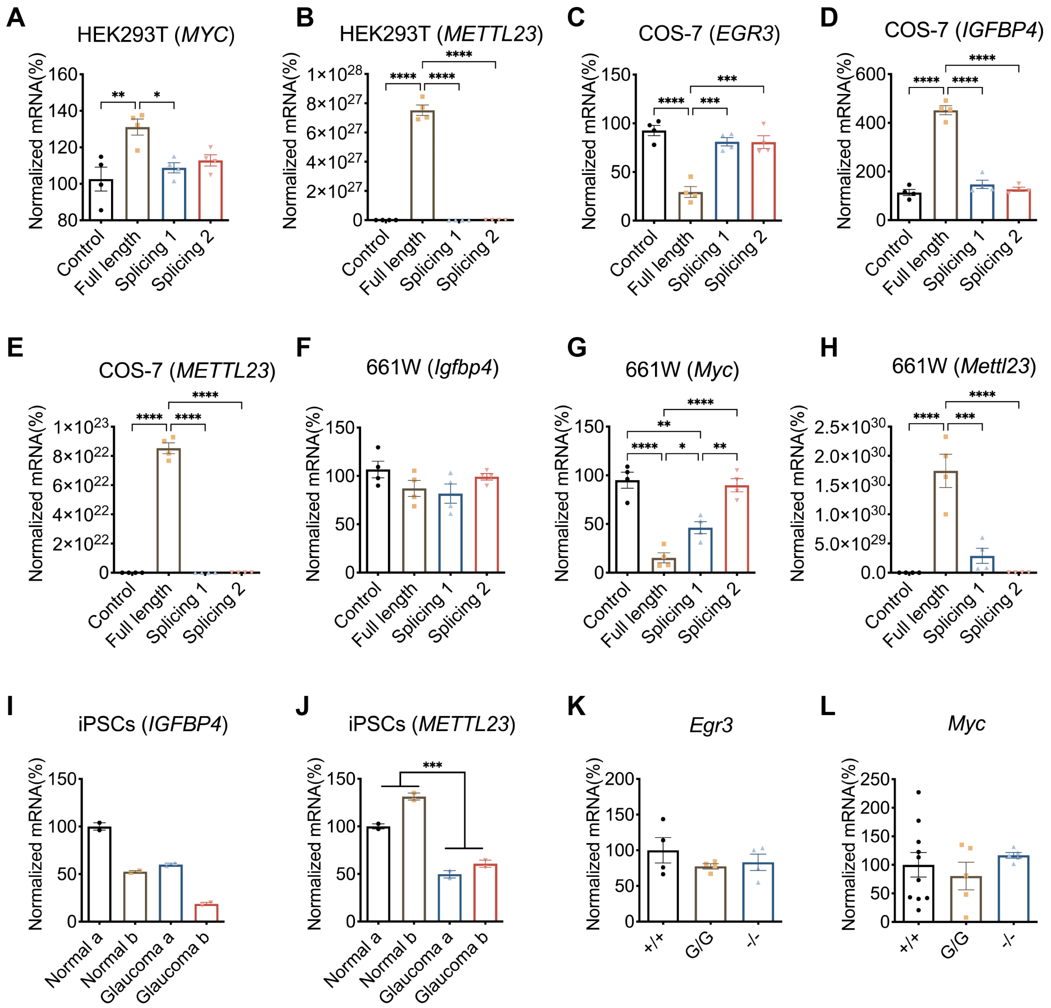

Supplemental Figure 10. Three CARM1-mediated estrogen receptor α target genes were not regulated by METTL23/Mettl23. The mRNA levels of *MYC* (**A**, **G**, **L**), *EGR3* (**C**, **K**), *IGFBP4* (**D**, **I**) and *METTL23* (**B**, **E**, **H**, **J**) genes were analyzed by RT-qPCR in both METTL23-full and METTL23-splicing1/splicing 2 transfected HEK293T cell (**A**, **B**), COS-7 cell (**C**, **D**, **E**), 661W (**F**, **G**, **H**), iPSCs (**I**, **J**), *Mettl23*$^{G/G}$ and *Mettl23*$^{-/-}$ (**K**, **L**). All data are presented as mean ± SEM. *: *P*<0.05, **: *P*<0.01,***: *P*<0.001, ****: *P*<0.0001 by one-way ANOVA followed by Tukey's multiple comparison test.

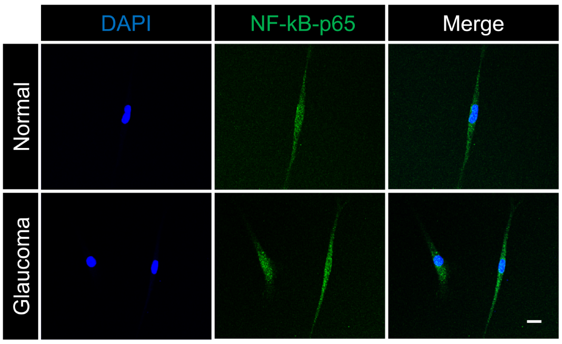

Supplemental Figure 11. Immunofluorescence staining for phospho-NF-κB–p65 (Ser536) in iPSC-RGCs. DAPI (blue) was used as a nuclear counterstain. Scale bars: 20 μm.

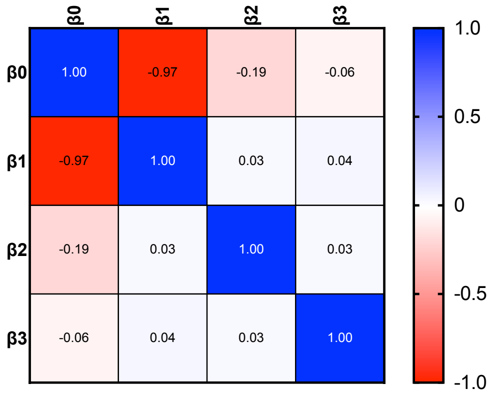

Parameter estimates	Variable	Estimate	Standard error	95% CI (profile likelihood)
β0	Intercept	-0.8485	0.2519	-1.345 to -0.3569
β1	Age	0.007159	0.003519	0.0002861 to 0.01409
β2	Female	0.07512	0.08237	-0.08630 to 0.2366
β3	METTL23 c.84+60delAT	0.9245	0.4462	0.07073 to 1.847

Odds ratios	Variable	Estimate	95% CI (profile likelihood)
β0	Intercept	0.4281	0.2606 to 0.6998
β1	Age	1.007	1.000 to 1.014
β2	Female	1.078	0.9173 to 1.267
β3	METTL23 c.84+60delAT	2.521	1.073 to 6.343

Sig. diff. than zero?	Variable	|Z|	P value	P value summary
β0	Intercept	3.369	0.0008	***
β1	Age	2.035	0.0419	*
β2	Female	0.9120	0.3618	ns
β3	METTL23 c.84+60delAT	2.072	0.0383	*

Supplemental Figure 12. Results of the multiple logistic regression to predict incident NTG. The values for female and METTL23 c.84+60delAT are taken as “0” if the particular factor is absent, and as “1” if it is present. β1= age, β2=female, β3= METTL23 c.84+60delAT.

References

1. McDonald KK, Abramson K, Beltran MA, Ramirez MG, Alvarez M, Ventura A, et al. Myocilin and optineurin coding variants in Hispanics of Mexican descent with POAG. *J Hum Genet.* 2010;55(10):697-700.
2. Tucker BA, Solivan-Timpe F, Roos BR, Anfinson KR, Robin AL, Wiley LA, et al. Duplication of TBK1 Stimulates Autophagy in iPSC-derived Retinal Cells from a Patient with Normal Tension Glaucoma. *J Stem Cell Res Ther.* 2014;3(5):161.
3. Hill JT, Demarest BL, Bisgrove BW, Su YC, Smith M, and Yost HJ. Poly peak parser: Method and software for identification of unknown indels using sanger sequencing of polymerase chain reaction products. *Dev Dyn.* 2014;243(12):1632-6.
